# Randomized controlled trial of neurologic music therapy in Parkinson’s disease: research rehabilitation protocols for mechanistic and clinical investigations

**DOI:** 10.1186/s13063-021-05560-7

**Published:** 2021-08-28

**Authors:** Isabelle Buard, Lucas Lattanzio, Rebekah Stewart, Sarah Thompson, Kristin Sjoberg, Karen Hookstadt, Meghan Morrow, Samantha K. Holden, Stefan Sillau, Michael Thaut, Benzi Kluger

**Affiliations:** 1 Department of Neurology, University of Colorado Denver, Fitzsimons Building, Mailstop F548, 13001 E. 17th Place, R24-002, Aurora, CO 80045 USA; 2 Rehabilitative Rhythms, Aurora, CO USA; 3 University of Colorado Hospital, Aurora, CO USA; 4 Faculty of Music, University of Toronto, Toronto, Canada; 5 Department of Neurology, University of Rochester Medical Center, Rochester, NY USA

**Keywords:** Magnetoencephalography, Motor cortical activity, Neuronal entrainment, Rehabilitation, Parkinson’s disease, Neurologic music therapy

## Abstract

**Background:**

Presently available medications and surgical treatments for Parkinson’s disease have limited effects on fine motor problems and often leave patients with significant fine motor disability. Standard of care occupational therapy (OT) yields low efficacy, potentially due to a lack of standard protocols. Neurologic music therapy (NMT) techniques, especially rhythmic auditory stimulation which relies on interaction between rhythm and movement, have shown to be effective in PD gait rehabilitation possibly through their reliance on neural pathways that are not affected by PD. Therapeutic instrumental music performance (TIMP) is one other NMT technique that holds promise but which mode of action and efficacy has not been investigated in PD yet.

**Methods:**

One hundred PD participants will be randomly assigned to receive 15 sessions of either TIMP with rhythm or TIMP without rhythm, standard of care OT, or to be waitlisted (control) over 5 consecutive weeks. Brain oscillatory responses will be collected using magnetoencephalography during an auditory-motor task to understand the underlying mechanisms. The Grooved Pegboard, the UPDRS III finger tap, and the finger-thumb opposition will be assessed to investigate clinical changes related to fine motor function. This project will also serve to confirm or refute our pilot data findings suggesting NMT relies on compensatory brain networks utilized by the PD brain to bypass the dysfunctional basal ganglia.

**Discussion:**

This study aims to use standardized TIMP and OT research protocols for investigating the neuronal pathways utilized by each intervention and possibly study their efficacy with respect to fine motor rehabilitation via a randomized control trial in the PD population.

**Trial registration:**

ClinicalTrials.gov NCT03049033. Registered on September 29, 2020

**Supplementary Information:**

The online version contains supplementary material available at 10.1186/s13063-021-05560-7.

## Administrative information

**Table:** 

{1} Title	Randomized Controlled Trial of Neurologic Music Therapy in Parkinson’s Disease: Research Rehabilitation Protocols for Mechanistic and Clinical Investigations
{2a} Trial Registration	ClinicalTrials.gov ID: NCT03049033
{2b} WHO Trial Registration	According to the World Health Organization Trial Registration Data Set guidelines, we have ensured all items to be included within this published protocol.
{3} Protocol Version	Colorado Multiple Institutional Review Board # 16-2308; Version 2 April 2020
{4} Funding	National Center for Complementary and Integrative Health (NCCIH) Grant Award #1K01AT009894-01A1 (PI: Buard). University of Colorado Denver Movement Disorders Center Pilot Grant (PI: Buard).
{5a} Author details	Isabelle Buard, PhD - Department of Neurology, University of Colorado Denver, Aurora, CO, USA Lucas Lattanzio, BA - Department of Neurology, University of Colorado Denver, Aurora, CO, USA Stefan Sillau - Department of Neurology, University of Colorado Denver, Aurora, CO, USA Samantha Holden - Department of Neurology, University of Colorado Denver, Aurora, CO, USA Benzi Kluger, MD MS - Department of Neurology, University of Rochester Medical Center Rochester, NY, USA Michael Thaut, PhD - Faculty of Music, University of Toronto, Canada Sarah Thompson, MT-BC, NMT - Rehabilitative Rhythms, Aurora, CO, USA Rebekah Stewart, MT-BC, NMT - Rehabilitative Rhythms, Aurora, CO, USA Kristin Sjoberg, MT-BC, NMT - Rehabilitative Rhythms, Aurora, CO, USA Karen Hookstadt, OT/R - University of Colorado Hospital, Aurora, CO, USA Meghan Morrow, OT/R - University of Colorado Hospital, Aurora, CO, USA
{5b} Name and contact information for the trial sponsor	Wen G. Chen, MMSc, PhD Acting Branch Chief, Basic and Mechanistic Research Branch Division of Extramural Research National Center for Complementary and Integrative Health (NCCIH), NIH, DHHS Phone: 301-451-3989 Email: chenw@mail.nih.gov NCCIH Web site: nccih.nih.gov
{5c} Role of sponsor	The sponsors play no role in study design, collection, management, analysis, interpretation of data, writing of the report, or the decision to submit the report for publication.

## Introduction

### {6a} Background and rationale

Parkinson’s disease [1] is the second most common neurodegenerative illness and affects 1% of people over age 50 and more than 10 million people worldwide [2]. Notable motor symptoms that manifest from PD are bradykinesia, rigidity, tremors, unnatural gait, and postural instability characterized by impairments in balance and coordination [3]. Besides gross motor symptoms, fine motor impairments in PD cause difficulties with everyday tasks such as writing, self-care, and fine object manipulation [4]. These activity limitations can lead to disability, social isolation, and a reduced quality of life [5]. Presently available medications and surgical treatments for PD have limited effects on fine motor problems, possibly because these symptoms are due to intrinsic dysfunction of the somatosensory cortex [6]. Advances in our understanding of the underlying neurophysiology have helped treat gross motor symptoms. Better evidence-based treatment strategies for PD-related fine motor dysfunction are clearly needed.

In a series of breakthrough studies, Michael Thaut and colleagues developed a series of evidence-based interventions collectively identified as neurologic music therapy (NMT), which includes techniques for gross motor neurorehabilitation that rely on interaction between rhythm and movement. Rhythmic auditory stimulation (RAS), one of these NMT techniques, has proven evidence of efficacy for gait rehabilitation in PD [7] and in stroke patients (now included in US VA and DoD as well as Canadian governmental guidelines). The pathological basal ganglia (BG) in PD brains leads to a reduced supply of internally generated movements. In contrast, externally cued movements (i.e., via a beat or a rhythm) during RAS sessions are instantaneously entrained to the period of a rhythmic stimulus possibly bypassing defective pallidocortical projections [8] via the lateral premotor cortex which receives sensory information in the context of externally guided movements [9]. The mechanism of action is called “rhythmic entrainment” where one system's motion or signal frequency entrains the frequency of another system. While published data suggest that RAS facilitates locomotor function in patients with PD [10, 11], effects of NMT techniques on fine motor function have not been investigated yet. Therapeutic instrumental music performance (TIMP) is another NMT technique which uses musical instruments in a therapeutic form to exercise and stimulate functional movement patterns [12]. In stroke patients, a TIMP-like intervention, called music-supported therapy, has shown motor benefits through movement exercises facilitated by musical instruments [13]. No evidence of TIMP-related rehabilitation in PD has been shown yet nor any evidence of how TIMP affects humans’ neurophysiology. Given the increasing interest related to music as a potential therapy for brain diseases, as well as new funding initiatives from the National Institute of Health, furthering our understanding via mechanistic clinical studies seems to hold enormous potential. Occupational therapy (OT) is the standard of care option when pharmacological interventions do not address fine motor impairments for PD patients. OT interventions typically include range of motion and strengthening of the extrinsic and intrinsic muscles, variable hand manipulation tasks, and coordination drills focusing on mass practice of designated movements. Unfortunately, these have a low impact, mainly due to patients’ compliance and lack of standardization.

It is known that neurons in the brain communicate with each other by firing at certain frequencies. Prior studies suggest impaired oscillatory activity in PD, such as excessive beta (15–30 Hz) synchrony [14] and impaired gamma activity [15]. Attempts to normalize beta synchrony in PD such as with deep brain stimulation surgery or dopaminergic medications are associated with improved motor symptoms [16], suggesting an interrelationship between altered brain rhythms and motor dysfunction in PD. In healthy controls, beta and gamma rhythms are suspected to play a role during rhythmic entrainment via a complex network system involving cortical and subcortical areas, such as the supplementary motor area, the parietal cortex and the basal ganglia [17, 18]. The cortical pathways involved in rhythmic entrainment of motor oscillations via NMT techniques are still unknown, and whether the PD brain mobilizes different networks during this process is still under investigation. The main hypothesis of this proposal is that cortical activity associated with fine motor control is impaired in PD and that NMT holds promise to normalize oscillatory activity via compensatory networks between the auditory and motor cortex. Bypassing the dysfunctional basal ganglia, NMT works to improve motor skills through neural entrainment. Regardless of the rehabilitative method used, this will also be the first study looking at the impact of fine motor training on neurophysiology.

### {6b & 7} Research objectives and comparators

The central goal for this study is to investigate the underlying networks used during TIMP and test the rehabilitative power of TIMP techniques for fine motor control in PD. Specifically, the study aims to demonstrate whether the use of musical instruments to specifically promote fine motor function allows differential mobilization of neuronal networks when they are combined with external rhythmic cueing. To allow for an accurate clinical comparator, a standardized OT research protocol will be tested as well.

## Methods

### {9} Study setting

Pre- and post-intervention testing will occur in the magnetoencephalography (MEG) area of the CU Neuromagnetic Lab within the Fitzsimons Building at the University of Colorado Anschutz Medical Campus. TIMP without external rhythm (TIMP-NR) and TIMP with external rhythm (TIMP-RHY) sessions will occur at the neurologic music therapists’ clinic whereas OT sessions will occur at an OT clinic. This study includes locations throughout Colorado for each of these therapy interventions.

### {15, 24 &26a} Recruitment and informed consent

Participants will be recruited from the Movement Disorders Center at University of Colorado Anschutz Medical Campus, from the community via advertisements, fliers, and through support group outreach. After a preliminary telephone screening, evaluation and consent will be obtained at a face-to-face interview between the subject and a member of the research team. Study details will be explained to the subject in a quiet room, without disturbance. Each subject will be asked to review the consent document, approved by the Colorado Multiple Institutional Review Board, and the University of Colorado Committee for the Protection of Human Subjects, which will include HIPAA authorization. Although this trial has minimal risk, subject harms fall under responsibility of the subject and their insurance, as indicated in the consent form.

### {26b} Additional consent provisions for collection and use of participant data and biological specimens

N/A

### {10} Eligibility criteria

Our inclusion criteria for the study include the following: (1) PD patients diagnosed using UK Brain Bank Criteria, (2) ages 45–85 at study entry, (3) provide independent consent, and (4) subjects with stable medications for at least 30 days will be included. Our exclusion criteria consist of the following: (1) features suggestive of other causes of parkinsonism, including cerebrovascular disease or history of major head trauma, (2) inability to move their fingers or hands, [19] Hoehn and Yahr stage 4 or higher, (4) ferrous metal implants which may interfere with the MEG data acquisition, (5) dementia according to the Mattis Dementia Rating Scale—Version 2 [20]. Cutoff score of ≤ 123 on the MDRS is proven to discriminate PD with from without dementia [21], (6) participants engaged in other research studies involving music, and (7) participants whose insurance does not cover OT costs or who have no insurance.

### {8 & 13} Trial design and timeline

**Fig. 1:**
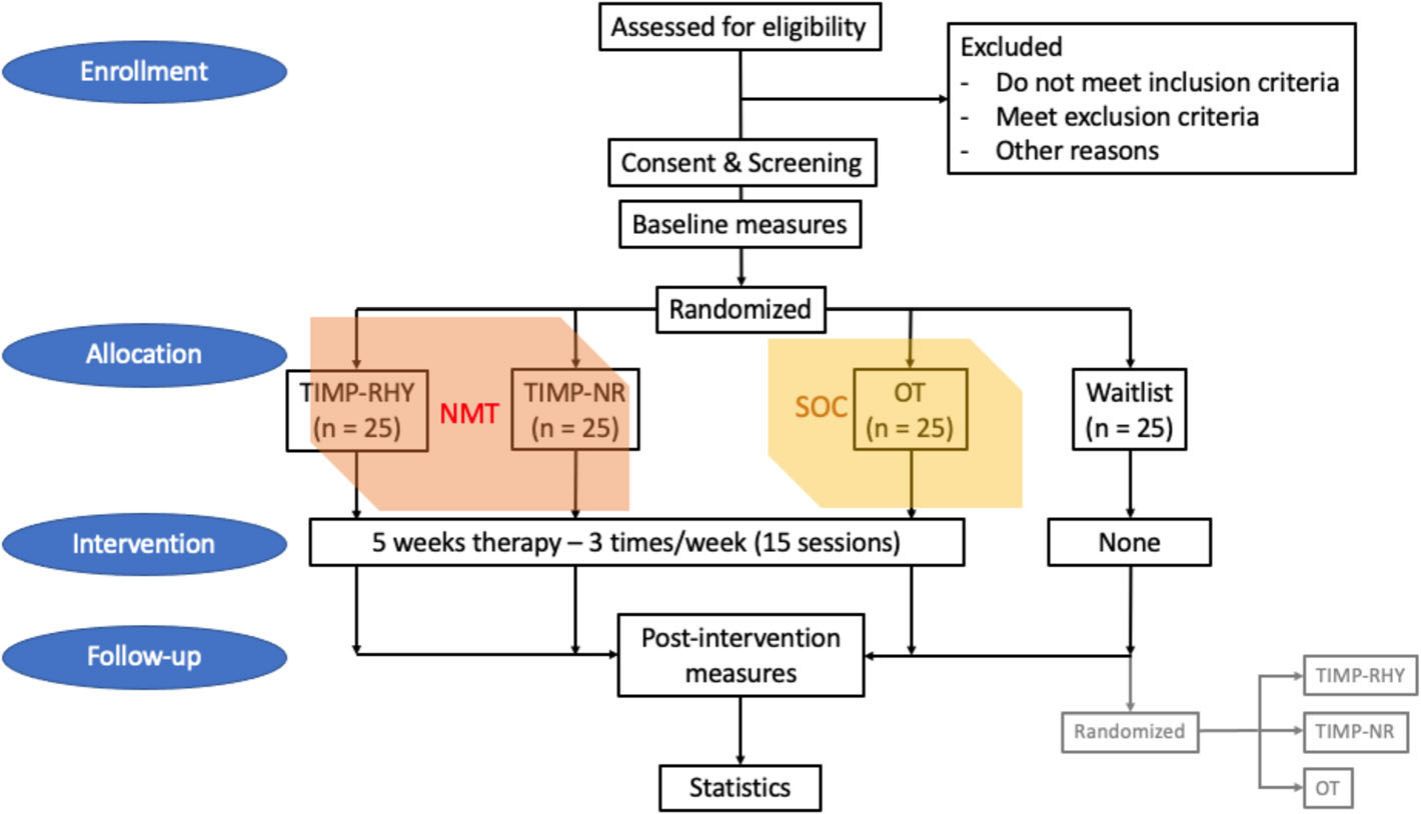
Study flowchart

After enrollment and screening assessment, 100 PD subjects will be randomly assigned (parallel assignment) in a 1:1:1:1 ratio to TIMP-RHY (25 people), or OT (25 people), or TIMP-NR (25 people) groups, or to wait for 5 weeks (Waitlist control group; 25 people). All PD subjects will undergo a magnetoencephalography (MEG) scan and a battery of tests before and after the TIMP-NR, TIMP-RHY, OT intervention, or twice separated by a 5-week period free of intervention (waitlist control group). First, to capture neural network and neurophysiological changes, participants will perform an MEG scan which engages them in an auditory-motor task. Then, clinical measures will be based on standardized PD neurologic assessments via the Unified Parkinson’s Disease Rating Scale [1], and fine motor assessments will include the Grooved Pegboard Test and finger-thumb opposition tests. Last, to capture changes in quality of life (QOL), we have included the 39-Item Parkinson's Disease Questionnaire (PDQ-39) [22]. The total duration of participation is 5–7 weeks (see the study flowchart in Fig. 1).

### {16a-c} Randomization and assignment of interventions

Group randomization is determined using a Microsoft Excel-based random number generated sequence which relates to one of four possible groups the subject can be randomized into. Subjects will be informed of the 4 possible group assignments. After initial screening, potential participants will be entered sequentially into the randomization list and therefore assigned to the intervention associated with their row. For scheduling and logistic purposes, subjects will learn of their group assignment once they are assigned a study ID and have been scheduled for their first research visit, which needs to occur within a week of the first intervention sessions. Subjects will typically be informed at least 2 weeks prior to starting on the study via email or telephone from our study coordinator. There is a second random number generated sequence for re-randomizing those who were initially randomized into the waitlist control group. This list re-randomizes those participants into either TIMP-RHY or TIMP-NR group or OT group after completing their post-waitlist visit. No data collection will occur for this second assignment.

### {17a-b} Blinding

Treatment assignment is blinded except for the clinical research coordinator, who is responsible for disseminating the group assignment from the randomization list. Treatment allocation is not blinded for the therapist and participant, as the nature of TIMP and OT therapy does not permit blinding. However, since music and occupational therapists will not collect any data, this should minimize bias. In addition, the participants are blinded to the main hypotheses of the study. Last, the PI, outcome assessors, and study statistician are blinded to all study conditions. No unblinding is permitted under any circumstance.

### {11a-d} Interventions

- *(i). TIMP*: Research participants will work with a bachelor’s or master’s level, board-certified MT, neurologic music therapist part of our team 3 times a week for 5 consecutive weeks. Based on the NMT principles, our TIMP interventions utilize a weighted keyboard to allow for auditory feedback of force exerted and will provide an appropriate amount of physical resistance against the finger strike. This offers opportunities for fine motor strength training in addition to working on dexterity. Castanets are being be used as well, for strengthening pincer grasp, a skill that helps turning pages in a book or picking up coins. Last, fine and gross motor warm-ups including range of motion exercises are included in each session.

**Table:** 

Identified needs of the population	Matched elements of the protocol
Bilateral movement	Warm up exercises Castanet playing Simultaneous bilateral piano playing
Finger isolation	Warm up exercises Multiple piano exercises (5-finger scales, 3-note arpeggios, Hanon-type exercises)
Finger strength	Use of a weighted keyboard or piano Resistance of castanets
Crossing midline	Use of full key range for distal and midline crossing
Range of motion	Transfer exercises (2c) Use of full key range for gross motor range of motion
Functional transfer	Object transfer task Improvisation piano task for self-cued movement without the motor learning element
Entrainment (for TIMP-RHY only)	Tempo assessment and use of metronome

The TIMP-NR research protocol uses musical instruments only as movement endpoints to provide auditory feedback for facilitating functional movement patterns [23]. The TIMP-RHY research protocol adds to the TIMP-NR an accompaniment with auditory rhythmic cues (via a metronome) to provide the critical feedforward information that creates anticipation, repetition, periodic stability (each cycle length consistent), and continuous time referencing to optimize trajectory, velocity, and acceleration (how much time has elapsed, how much time is left during the movement). On the first TIMP-RHY therapeutic session, a baseline tempo for the TIMP exercise (4a-d) will be assessed for each participant using a metronome. Then, the baseline tempo will be increased by 5% each week up to 180 bpm. Full TIMP-NR and TIMP-RHY research protocols are available for reference in the Supplementary documents section. Importantly, TIMP-RHY relies on external cueing for movement generation whereas TIMP-NR requires internally generated movements. Of note, patterned sensory enhancement (PSE), another sensorimotor rehabilitation NMT technique, is briefly utilized in the TIMP-RHY protocol and uses “musical patterns to assemble single, discrete motions (e.g., arm and hand movements during reaching and grasping), into functional movement patterns and sequences” [23]. Information regarding these techniques is available at NMTAcademy.co.

- *(ii). Standard of care OT*: Participants who are randomized into the OT group will receive standard of care OT sessions, as prescribed by the patient’s primary neurologist. Research participants will work with one of the certified occupational therapists on our team, who all have expertise in PD and/or other neurological disorders. The occupational therapists will provide care 3 times per week for 5 consecutive weeks using the OT research protocol to provide a standard of care control matched for time with music therapist (full OT research protocol available in the Supplementary documents). Occupational therapists have robust experience in neurological recovery and are well versed in neurological re-education after the onset of a neurological injury. By incorporating their background knowledge on how the basal ganglia and proprioceptive tracks influence movement and how that movement impacts functional abilities, each individual’s engagement in this research study will be facilitated and monitored. Occupational therapists use functional activities and the use of daily self-care tasks and occupations to improve performance and independence in each individual’s unique daily roles (home, community, work and/or school). Therefore, the OT protocol was developed focusing on aerobic, proprioceptive, and coordination skills required to succeed in any daily routine.

In the case of any missed intervention session, the assigned therapist and the clinical coordinator will be in contact with the participant to schedule a make-up session, which will also help ensuring trial adherence.

No protocol discontinuation or modification is expected, as delineated in our protocol and consent form.

Concomitant care will be allowed except changes in dopamine replacement medications and interventions targeting specifically fine motor skills.

### {33} Plans for collection, laboratory evaluation, and storage of biological specimens for genetic or molecular analysis in this trial/future use

N/A

### {12} Outcomes

Primary mechanistic outcome for this study will be neurophysiology. We aim to uncover the underlying pathways utilized by NMT in the PD brain. Neurophysiologic data will be collected via MEG before and after any therapy or twice separated by a five week interval. We will be using a whole head neuromagnetometer (4D Neuroimaging) with an array of 248 sensors at a 678.17 Hz sampling rate and an acquisition bandwidth of 0.1–200 Hz during an auditory-motor task. Participants will be presented with 6 sequences of 30 ms acoustic burst stimuli (2000 Hz, intensity of 70 dB above subjective threshold) presented at 1 Hz. Each sequence will last 30 s with a 5-s break (180 total stimuli). They will be asked to tap their dominant index finger with the stimuli. After MEG data cleaning and preprocessing, time-frequency transformation and source localization will allow to measure relative beta and gamma oscillatory power in our regions of interest. We will evaluate group changes in directional functional connectivity between auditory, motor, and other regions of interest in the frequency domain using Granger causality.

Our primary motor outcome for this study will be finger dexterity. Similarly to our mechanistic outcome, will record changes in the total score on the Grooved Pegboard Test (GPT), which is a manipulative dexterity test consisting of 25 holes with randomly positioned slots [24] commonly used as a test of fine motor performance [25]. As the pegs must be rotated into position to be successfully placed, the GPT adds a dimension of complexity not found in other motor tasks and thus has been found to be a sensitive instrument in detecting general slowing due to medication or disease progression. In PD, the Grooved Pegboard Test has also been used extensively for identifying lateralized impairment [26] and as a motor outcome of clinical trials [27]. The time taken to complete the GPT with each hand is the score to be used in this application. Reliability for the test is good. Secondary motor outcomes designated for this study also assess fine motor abilities and will include the score on the finger-thumb opposition assessment (derived from the Neurological Evaluation Scale [28];) and the score from the finger tapping test on the UPDRS part III.

Quality of life (QOL) outcome measures will include the 39-Item Parkinson's Disease Questionnaire (PDQ-39) [22] and the Clinical Global Impression—Improvement Scale (CGI-I) to better define meaningful self-report and clinical changes. As an exploratory QOL outcome, we will include the Hospital Anxiety and Depression Scale (HADS) [29, 30] to assess for potential improvements in depressive symptoms and anxiety, which may improve with fine motor therapies.

### {18a-b, 19, 29} Data collection, management, and accessibility

MEG data collection is under the responsibility of a trained neurophysiology specialist to ensure data quality and assessment of unplanned data contamination. Motor scales and surveys are being collected by the study investigator who has been thoroughly trained by clinical and research colleagues during past clinical trials. Each measure and scale used in this study is widely used in the research community and has proven reliability and validity in the scientific literature. After collection, data is entered into the local Research Electronic Data Capture (REDCap) database, and data entry is verified by a research assistant non-affiliated to the study. Only complete sets of data (pre/post) will be included in the analysis. Study PI and statistician will have access to the final trial dataset.

### {14, 20a-c} Statistical methods and sample size

To address our primary mechanistic question, group comparison of mean Granger spectra changes using a linear mixed model controlling for sex and age will be analyzed during cued rhythmic tapping between regions of interest (ROIs: auditory, motor, precuneus, supramarginal gyrus and others) and corrected for multiple comparisons using the FDR method on the overall set of comparisons, *q* < 0.1. Statistically significant changes in Granger causality between ROIs are expected in the TIMP-RHY group but not in the OT or TIMP-NR groups due to the reliance on other motor networks, specifically those affected by PD pathology during fine motor training. A sample size per group of 25 subjects will allow us to determine between group differences with an effect size of 0.8 standard deviations/mean with 80% power and 0.05 significance, which is comparable to other studies examining neurophysiologic mechanisms of therapeutic interventions [31, 32] including similar studies using Granger causality as main outcomes in PD [33, 34]. We will perform exploratory analyses using linear regression to determine if any particular Granger feature is (1) associated with sex, age, or disease duration or severity and (2) more or less prone to motor function benefits. Based on our hypothesis, we predict fine motor training during TIMP-RHY, TIMP-NR, and OT will increase cortical motor beta and normalize gamma oscillations during the auditory-motor task in the MEG, although changes are expected to be stronger in the TIMP-RHY than in the OT and TIMP-NR groups. A 3 × 2 (group by frequency) mixed model ANOVA will be assessed to test this prediction.

We will address our clinical research question by determining whether there is a significant change in fine motor skills following TIMP-RHY, TIMP-NR, and OT due to fine motor training compared to the non-therapy condition. We predict that TIMP-RHY will decrease mean GPT scores to a higher extent than the TIMP-NR and the OT groups. We will use an ANOVA F-test model to test this prediction on the mean GPT score change as well as pairwise contrasts with Tukey-Kramer correction for multiple comparisons to assess where the differences are. We do not expect such changes in the waitlist group due to the absence of fine motor strengthening. Given the large effect size of 0.8 sd/mean seen in other PD studies using the GPT grooved pegboard test as main outcome [35, 36], we anticipate that our sample size of 25 per group will allow us to determine a group difference of 15% decreased time using the dominant hand and to be comparable to published data [37]. We will also perform exploratory analyses using regression modeling to assess whether (1) sex, age, or disease severity and (2) cortical motor beta power changes are correlated with fine motor tests performance.

We will address our QOL research question by assessing changes in the PDQ-39 and CGI-I scales. As fine motor skills may improve, we expect a decrease in mean total scores (the higher the scores the higher the problems with quality of life, depression/anxiety and how things have changed) after a 5-week TIMP-RHY session and to a lower extent after OT or TIMP-NR sessions. We do not expect a change of QOL mean scores in the waitlist group.

Only complete sets of data (including both pre and post) will be included in the analysis. No imputation or other methods for handling missing data will be used during the analysis process.

No interim or subgroup analyses will be performed.

### {5d, 21, 22, 23 & 25} Oversight and monitoring

This clinical trial, protocol, and consent form are approved by the Colorado Multiple Institutional Review Board, COMIRB # 16-2308. Amendments are submitted to COMIRB for approval. Important protocol modifications will be communicated to the sponsor for approval.

A physician external to the research team (S. K. H.) provides oversight as our data monitoring committee and is conducting a comprehensive review that occurs bi-annually. Any adverse events, regardless of the occurrence’s relationship to the study, will be documented and reported to the PI without indicating the location of the event, in order to maintain PI blinding. The level of severity and prospective actions are determined by the PI. Serious adverse events are released to COMIRB. Per department standards, routine auditing will occur to ensure all measures and standard operating procedures are followed as outlined in the protocol.

Considering the different stages of this trial, our team members are assigned to different tasks: recruitment (2 staff members), scheduling (study coordinator), randomization and statistical analyses (study statistician), consent process (study coordinator), baseline and post-intervention visits (study PI), and data management (outcome assessor).

### {31a,c & 32} Dissemination

De-identified data will be made available to the scientific community upon request. This data sharing will include motor tests, questionnaires, and MEG raw data. Study results will lead to public disclosure but cannot be traced back to the individual participants who took part in this study, as delineated in the consent form. We also plan to disseminate study results through peer-reviewed journal publications and conference presentations. Last, we would like to make the TIMP and OT protocols available to the community and to the World Federation for Neuro-Rehabilitation to encourage their use in clinics, with the potential long-term goal of making them standard practice if results from this trial are conclusive.

### Retention plan

We suspect that subject’s motivation and assiduousness might decline during the course of the 5 weeks. To prevent this, we will follow-up with the participants on a weekly basis via phone call to ensure proper completion of all the sessions. We will offer to arrange free rides via Access-a-Ride from participants’ homes to the therapy offices. If needed, we will modify some exercises to prevent possible patient difficulties with the current protocol.

### {30} Provisions for post-trial care

N/A—no harm is expected from trial participation.

## Discussion

While this project aims to test standardized clinical symptomatic approaches for PD rehabilitation, protocols created for this study only have a research value. They should not be used as clinical training manuals for music, occupational, or other therapists. However, exploring the feasibility aspect of these interventions in clinical or research settings as a research goal is highly encouraged. In this case, reaching out to the corresponding author may be helpful.

The mechanistic data provided with this trial may inform future research leading to treatment and rehabilitative interventions for patients with basal ganglia disorders and other neurologic diseases. In addition, this line of innovative research related to music and health will hopefully further our knowledge of the neural processes utilized by music.

It is essential to note that board-certified music therapists administering TIMP sessions will have completed the required NMT training and are in compliance with the NMT Academy requirements for utilizing NMT techniques. These music therapists have also satisfied research standards by demonstrating competency in facilitating the research protocol and completed research related training (i.e., CITI trainings).

### Trial status

Protocol version September 29, 2020. Recruitment started in February 2019, and the final participants are expected to complete their assessments at the end of 2023.

## Supplementary Information

**Additional file 1.** TIMP-RHY research protocol.

**Additional file 2.** TIMP-NR research protocol.

**Additional file 3.** OT research protocol.

## Data Availability

The raw data collected during this trial will be made available by the authors, without undue reservation.
