# Supplementary material for: Randomized controlled trial of neurologic music therapy in Parkinson’s disease: research rehabilitation protocols for mechanistic and clinical investigations
Source: Trials. 2021 Aug 28;22:577. doi: 10.1186/s13063-021-05560-7 (PMC8403394; doi:10.1186/s13063-021-05560-7)
Supplement: Supplementary file 1 — Additional file 1. TIMP-RHY research protocol. [file 13063_2021_5560_MOESM1_ESM.docx]

# TIMP-RHY RESEARCH PROTOCOL

1. **Greet participant, caregiver**

1. **DEXTERITY EXERCISES USING FUNCTIONAL MOVEMENTS (PSE)**

- 1. **RHYTHMIC CUEING OF HAND/FINGER MOVEMENTS**

**i.** Set metronome at 75bpm (week 1), 79bpm (week 2), 83bpm (week 3), 87bpm

(week 4), 91bpm (week 5) and cue with autoharp

**ii.** Open and close hands (large opening); both hands moving together – 10 repetitions


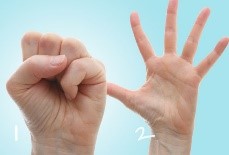


**iii.** pronation/supination (complete turns); both hands moving together – 10 repetitions


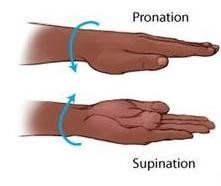


- 1. **RHYTHMIC CUEING OF FINGER-THUMB OPPOSITION MOVEMENTS (TOUCHING THUMB**
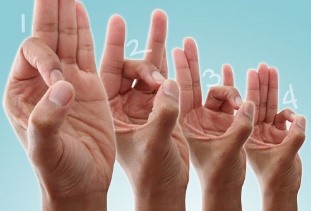
**SEQUENTIALLY WITH OTHER 4 FINGERS)**

Set metronome at 100bpm (week 1), 105bpm (week

2), 110bpm (week 3), 115bpm (week 4), 120bpm (week 5) and cue open and shut with autoharp; both hands move together 3x without pause

- 1. **RHYTHMIC CUEING OF HAND TO OBJECT MANIPULATIONS:**

**REACHING, GRASPING, HOLDING, LIFTING OBJECTS**

- - 1. Have participant sit in front of small table with 3 different objects; a cup, a spoon, and a coin. Items will be picked up, transferred, and set down sequentially: Reach, Grasp, Lift, Transfer, Let go and Bring arm back.

- - 1. Sessions 1-5: **Midline transfer to matching side**


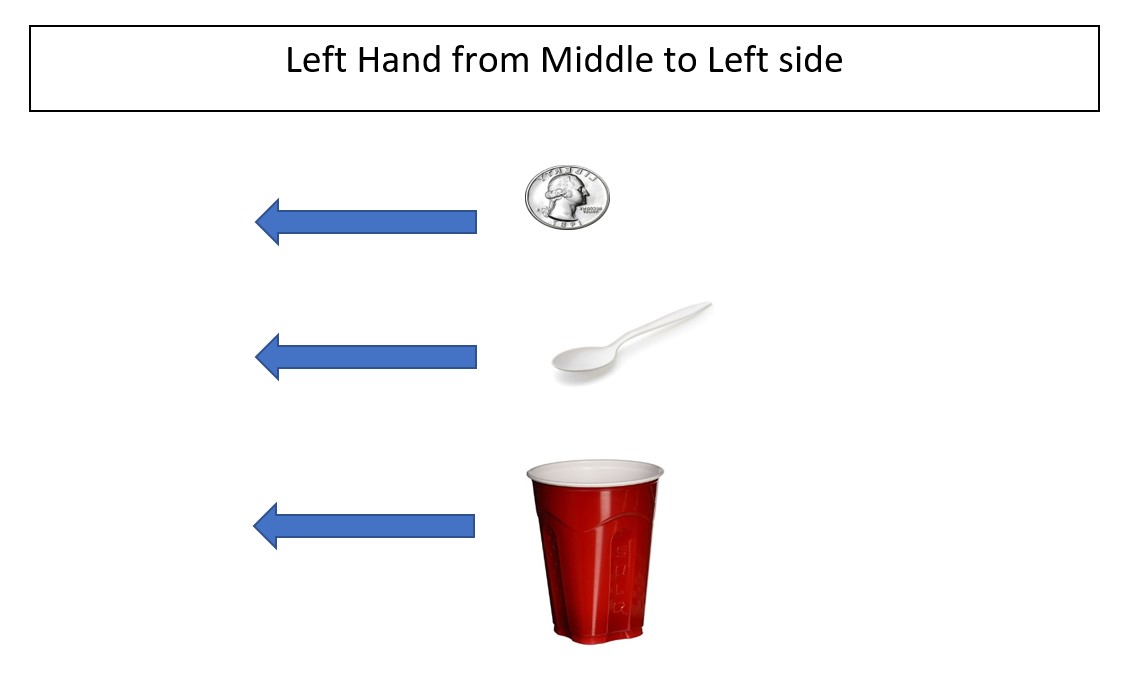

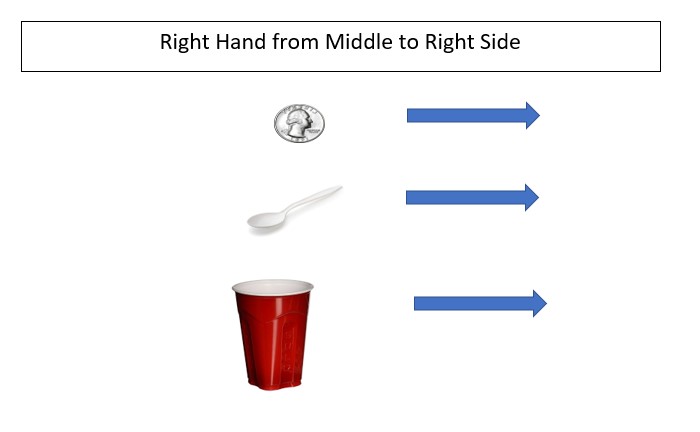


**iii.** Sessions 6-10: **Midline transfer to opposing side**


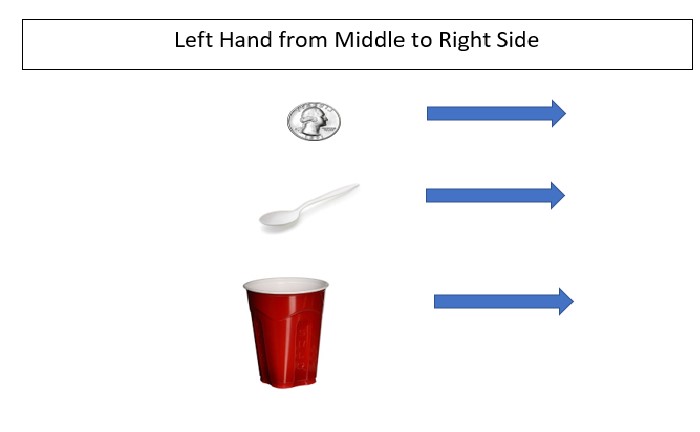

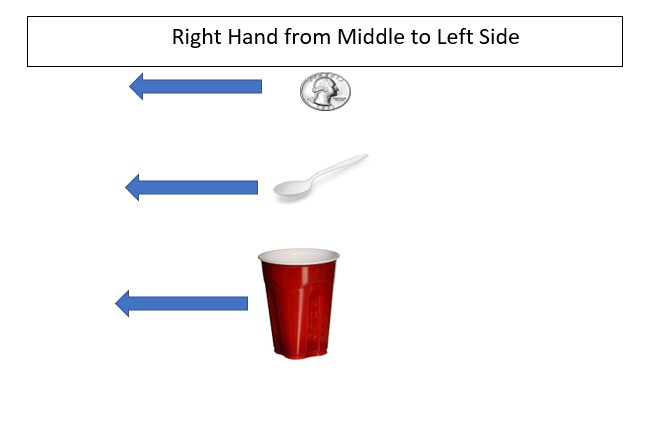


**iv.** Sessions 11-15: **Opposing side transfer across to matching hand side**


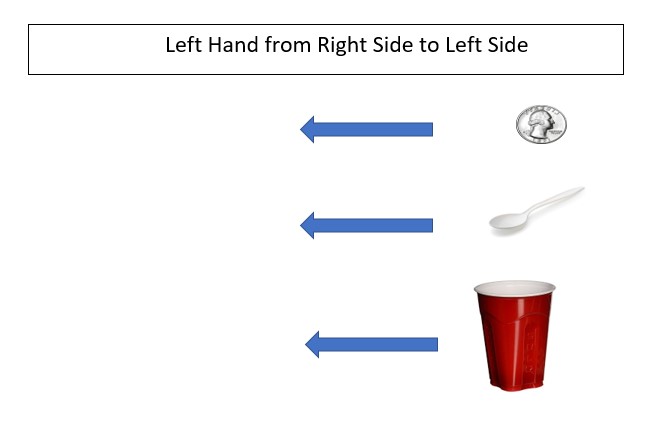

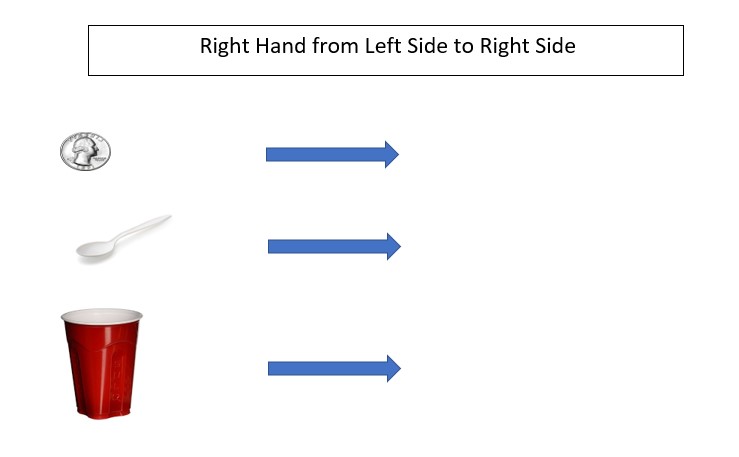


**v. Set metronome to 80 bpm and then adjusted only if that is too fast. (The at-home exercise is at 80bpm.) Tempo is increased by 5% each week.**

- - 1. Tempo ______(week 1) ______(week2) ______(week 3)

______(week4) ______(week 5)

- - 1. Facilitate exercises with autoharp. Chord Per Beat Progression for 2 bars of 6/8 is as follows: I-I-I-ii-ii-IV-I-I-I-V-V-V
    2. Transfer each object successfully one time with RUE and LUE


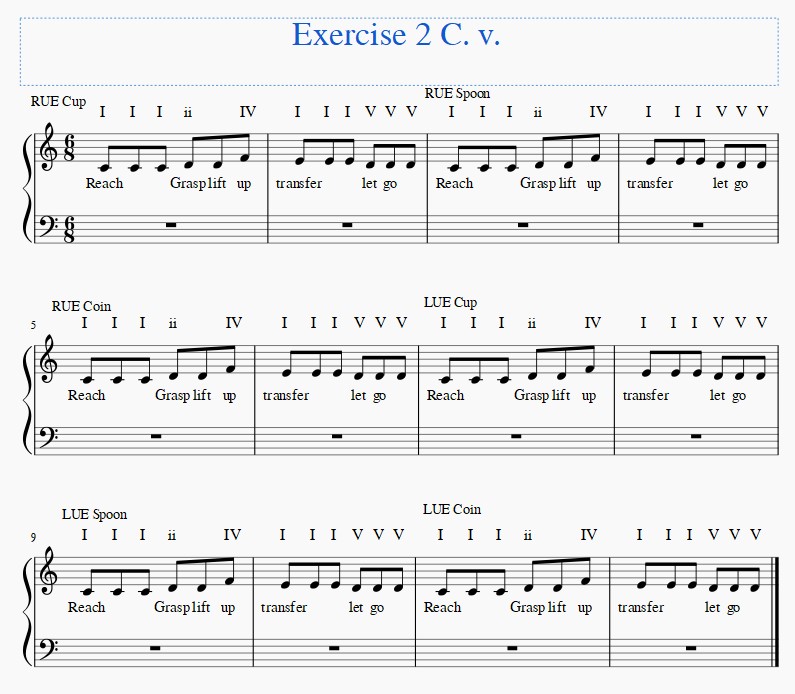


1.
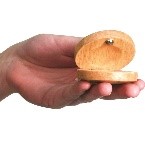
**CASTANET PLAYING EXERCISES WITH RHYTHMIC**

**HARMONIC CUEING**

- - 1. Give participant castanets, one in each hand.
    2. Have participant show you how fast they are going to play. Using the keyboard, guitar or autoharp, match the participant’s tempo.
    3. Set timer for 3-4 minutes and then play with participant
    4. Add in preferred song or set of songs that total 3-4 minutes of continuous playing.
    5. Participant starts with both hands playing castanets simultaneously.
    6. Halfway through switch to alternating hands every 2, 4, or 8 beats

1. **DEXTERITY EXERCISES ON KEYBOARD (TIMP)**

**! Tempo is increased by 5% each week !**

- 1. For session number one, assess client ability on each scale exercise and find optimal tempo for each exercise. Start metronome at 60bpm, increase by 10 bpm until pt. unable to complete exercise successfully, and decrease by 5bpm until consistent success is reached. Note bpm in assessment for each exercise.

**DO NOT START AT OR INCREASE TO ABOVE 180BPM FOR ANY EXERCISE**

- 1. **FINGER ADJACENT SCALES IN DIFFERENT FINGER COMBINATIONS:**

Repeat each exercise x1 100% success rate each hand (or more if extra time)

- - 1. Set metronome first. Have participant play scales. Repeat each exercise x1 100% success rate each hand.

Starting Pitch- Middle C (C4) on RH and C below middle C (C3) on LH

| RH 1-2-3-4-5, 5-4-3-2-1 | LH 5-4-3-2-1, 1-2-3-4-5 |
| --- | --- |
| RH Tempo week 1: | LH Tempo week 1: |
| RH Tempo week 2: | LH Tempo week 2: |
| RH Tempo week 3: | LH Tempo week 3: |
| RH Tempo week 4: | LH Tempo week 4: |
| RH Tempo week 5: | LH Tempo week 5: |


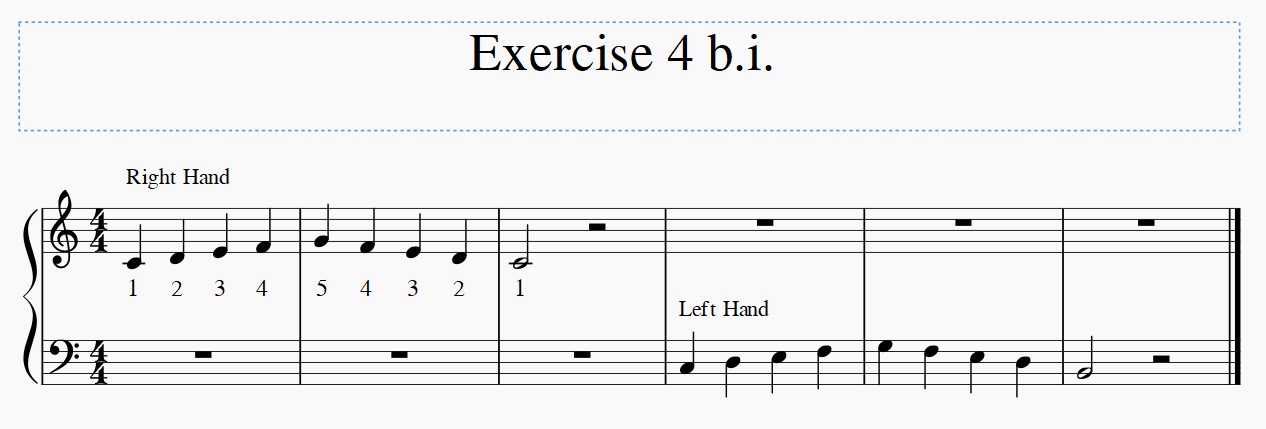


Right hand


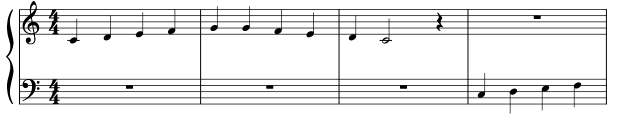


Left hand


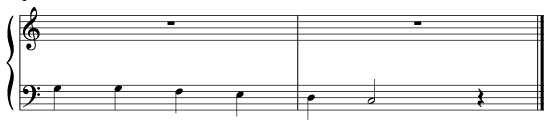


- - 1. Set metronome first. Have participant play arpeggios. Repeat each exercise x 1 100% success rate each hand (or more if extra time).

Starting Pitch- Middle C (C4) on RH and C below middle C (C3) on LH

| RH 1-3-5, 5-3-1 | LH 5-3-1, 1-3-5 |
| --- | --- |
| RH Tempo week 1: | LH Tempo week 1: |
| RH Tempo week 2: | LH Tempo week 2: |
| RH Tempo week 3: | LH Tempo week 3: |
| RH Tempo week 4: | LH Tempo week 4: |
| RH Tempo week 5: | LH Tempo week 5: |


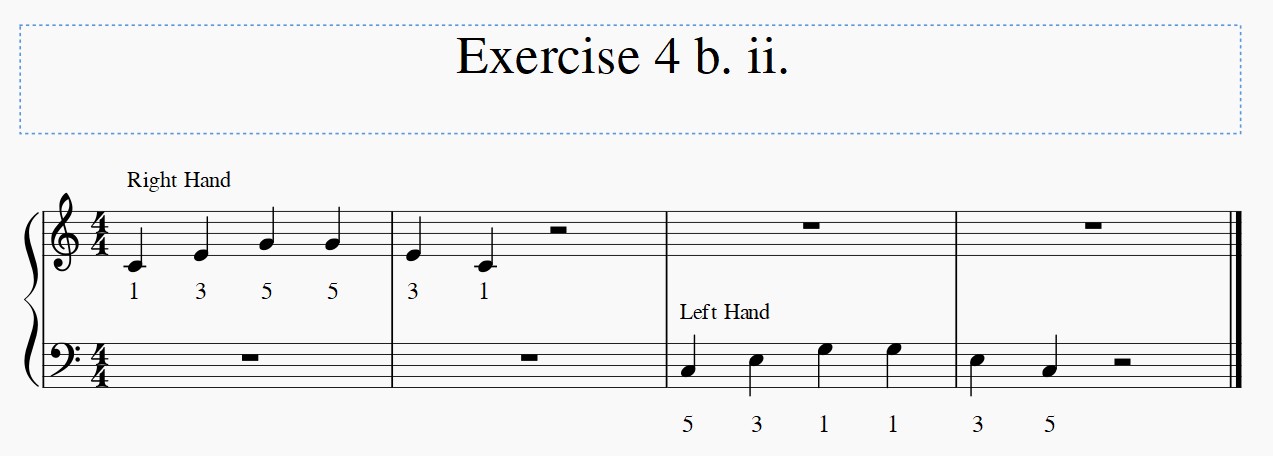


- - 1. Set metronome first. Have participant play alternating pattern. Repeat each exercise x 1 100% success rate each hand (or more if extra time). Starting Pitch- Middle C (C4) on RH and C below middle C (C3) on LH


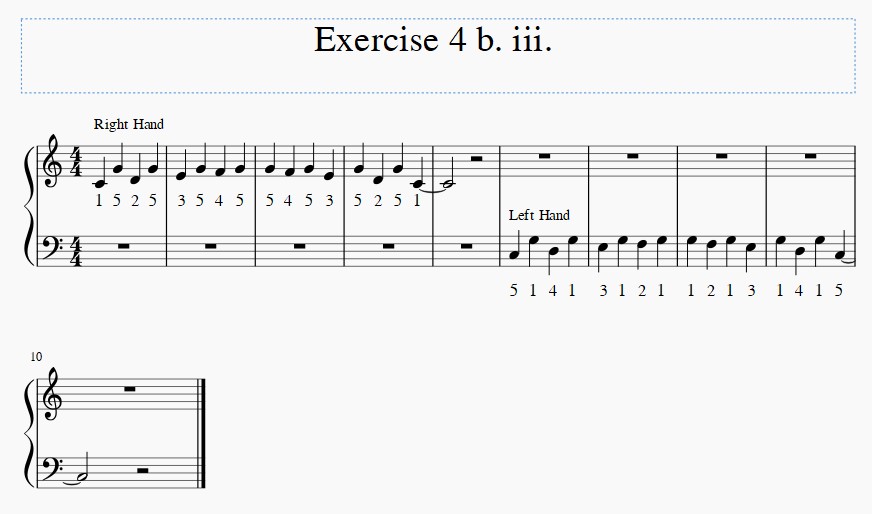


| RH 1-5-2-5-3-5-4-5, 5-4-5-3-5-2-5-1 | LH 5-1-4-1-3-1-2-1, 1-2-1-3-1-4-1-5 |
| --- | --- |
| RH Tempo week 1: | LH Tempo week 1: |
| RH Tempo week 2: | LH Tempo week 2: |
| RH Tempo week 3: | LH Tempo week 3: |
| RH Tempo week 4: | LH Tempo week 4: |
| RH Tempo week 5: | LH Tempo week 5: |

- 1. **SCALES PLAYING IN DIFFERENT OCTAVE REGIONS TO TRAIN FOR**

**FLEXIBILITY AND DISTAL MUSCLE INVOLVEMENT**

1. Starting Pitch RH- C6, One scale per octave, move down keyboard for 4 octaves. Starting Pitch LH- C2, One scale per octave, move up keyboard for 4 octaves.
2. ii. Set metronome first. Each exercise x 1 100% success rate per hand (or more if extra time).

| RH 1-2-3-4-5, 5-4-3-2-1 | LH 5-4-3-2-1, 1-2-3-4-5 |
| --- | --- |
| RH Tempo week 1: | LH Tempo week 1: |
| RH Tempo week 2: | LH Tempo week 2: |
| RH Tempo week 3: | LH Tempo week 3: |
| RH Tempo week 4: | LH Tempo week 4: |
| RH Tempo week 5: | LH Tempo week 5: |


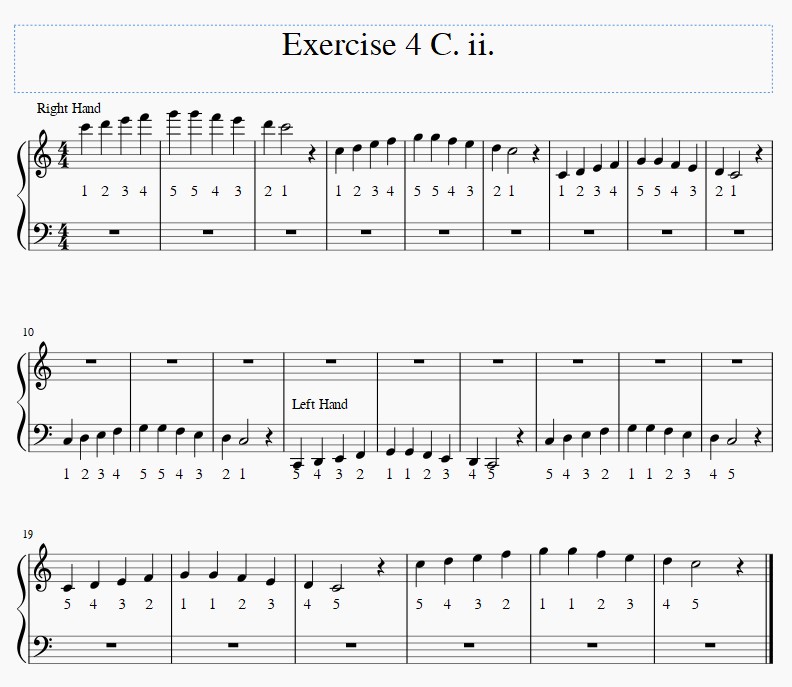


- - 1. Set metronome first. Each exercise x1 100% success rate per hand (or more if extra time).

Starting Pitch RH- C6, One scale per octave, move down keyboard for 4 octaves.

Starting Pitch LH- C2, One scale per octave, move up keyboard for 4 octaves.

| RH 1-3-5, 5-3-1 | LH 5-3-1, 1-3-5 |
| --- | --- |
| RH Tempo week 1: | LH Tempo week 1: |
| RH Tempo week 2: | LH Tempo week 2: |
| RH Tempo week 3: | LH Tempo week 3: |
| RH Tempo week 4: | LH Tempo week 4: |
| RH Tempo week 5: | LH Tempo week 5: |


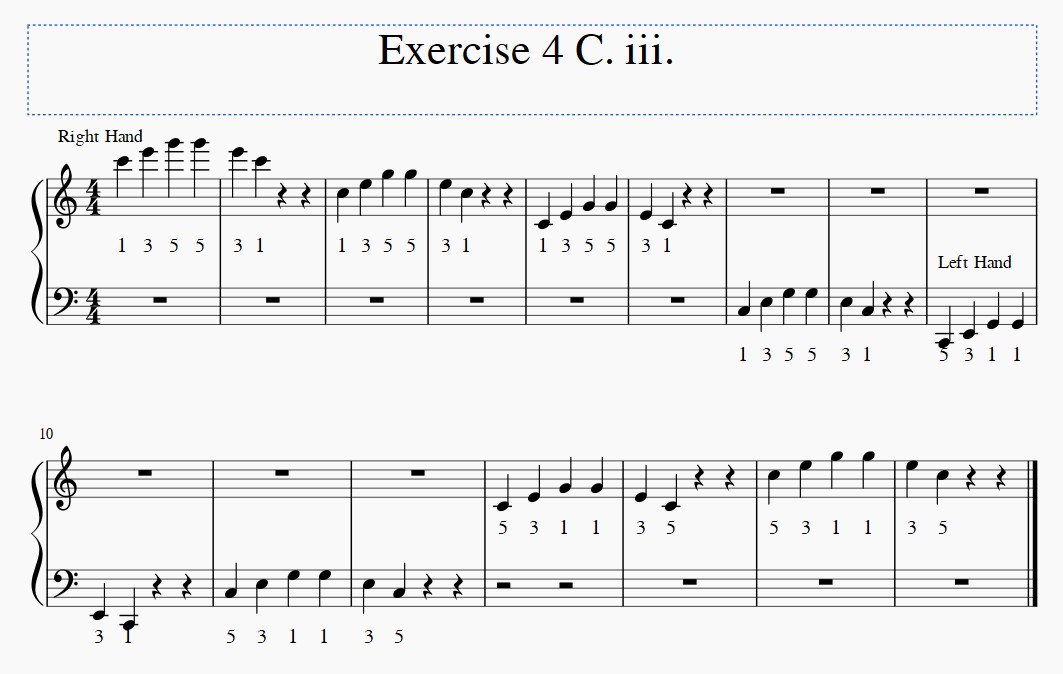


- - 1. Set metronome first. Each exercise x1 100% success rate per hand (or more if extra time).

Starting Pitch RH- C6, One scale per octave, move down keyboard for 4 octaves.

Starting Pitch LH- C2, One scale per octave, move up keyboard for 4 octaves.

| RH 1-5-2-5-3-5-4-5, 5-4-5-3-5-2-5-1 | LH 5-1-4-1-3-1-2-1, 1-2-1-3-1-4-1-5 |
| --- | --- |
| RH Tempo week 1: | LH Tempo week 1: |
| RH Tempo week 2: | LH Tempo week 2: |
| RH Tempo week 3: | LH Tempo week 3: |
| RH Tempo week 4: | LH Tempo week 4: |
| RH Tempo week 5: | LH Tempo week 5: |


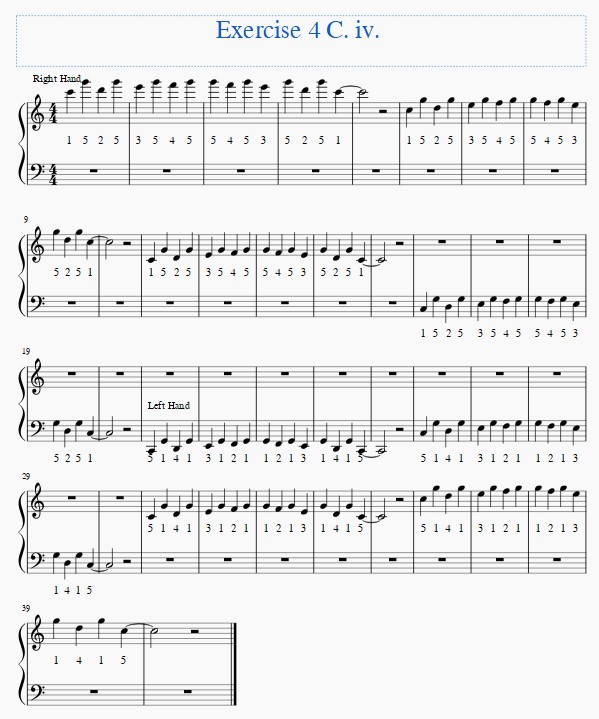


- 1. **SCALES PLAYING IN 3 HAND COMBINATIONS: ALTERNATING, SEQUENTIAL, AND SIMULTANEOUS:**
     1. Each exercise x1 100% success rate per hand (or more if extra time).

Alternating Starting Pitch- RH C5; Starting Pitch- LH C3

| LH 5-4-3-2-1, 1-2-3-4-5 | RH 1-2-3-4-5, 5-4-3-2-1 |
| --- | --- |
| RH Tempo week 1: | LH Tempo week 1: |
| RH Tempo week 2: | LH Tempo week 2: |
| RH Tempo week 3: | LH Tempo week 3: |
| RH Tempo week 4: | LH Tempo week 4: |
| RH Tempo week 5: | LH Tempo week 5: |


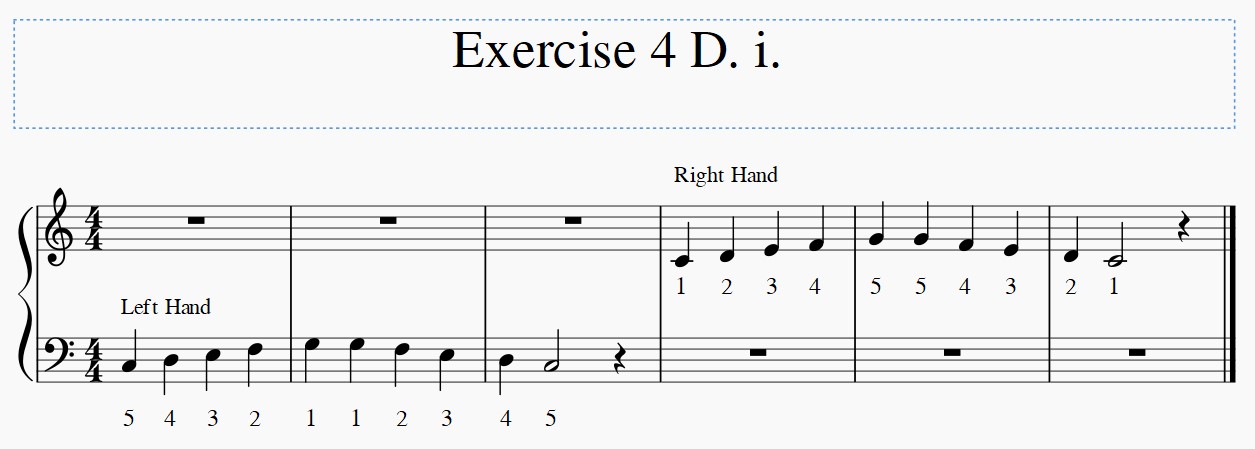


- - 1. Each exercise x1 100% success rate per hand (or more if extra time). Simultaneous Starting Pitch- RH C5; Starting Pitch- LH C3

| RH 1-2-3-4-5, 5-4-3-2-1 | LH 5-4-3-2-1, 1-2-3-4-5 |
| --- | --- |
| RH Tempo week 1: | LH Tempo week 1: |
| RH Tempo week 2: | LH Tempo week 2: |
| RH Tempo week 3: | LH Tempo week 3: |
| RH Tempo week 4: | LH Tempo week 4: |
| RH Tempo week 5: | LH Tempo week 5: |


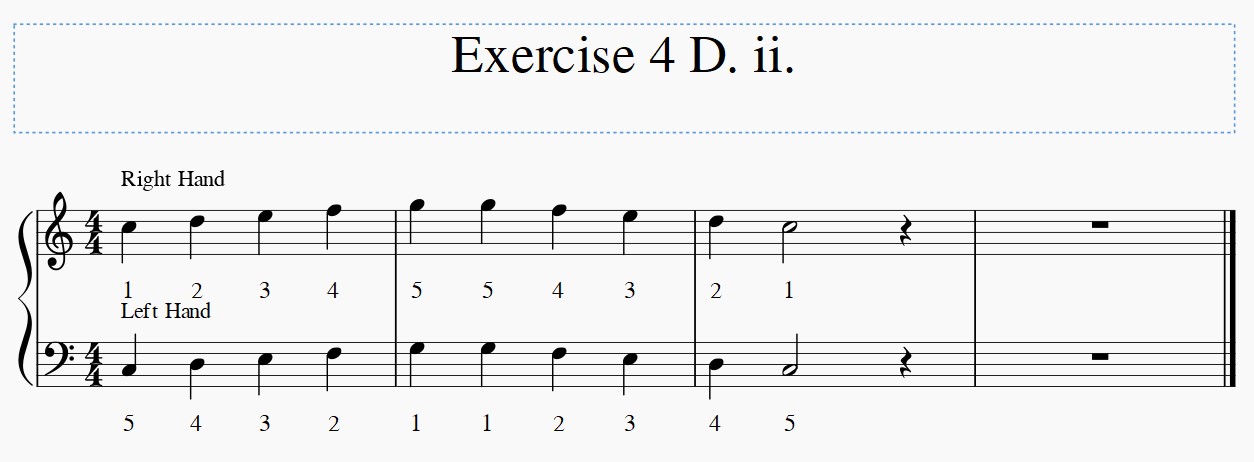


- - 1. Each exercise x1 100% success rate per hand (or more if extra time).

Sequential Starting Pitch- LH C2, RH C3; Ending pitch- RH C7, LH C6

| LH 5-4-3-2-1, 1-2-3-4-5 | RH 1-2-3-4-5, 5-4-3-2-1 |
| --- | --- |
| RH Tempo week 1: | LH Tempo week 1: |
| RH Tempo week 2: | LH Tempo week 2: |
| RH Tempo week 3: | LH Tempo week 3: |
| RH Tempo week 4: | LH Tempo week 4: |
| RH Tempo week 5: | LH Tempo week 5: |


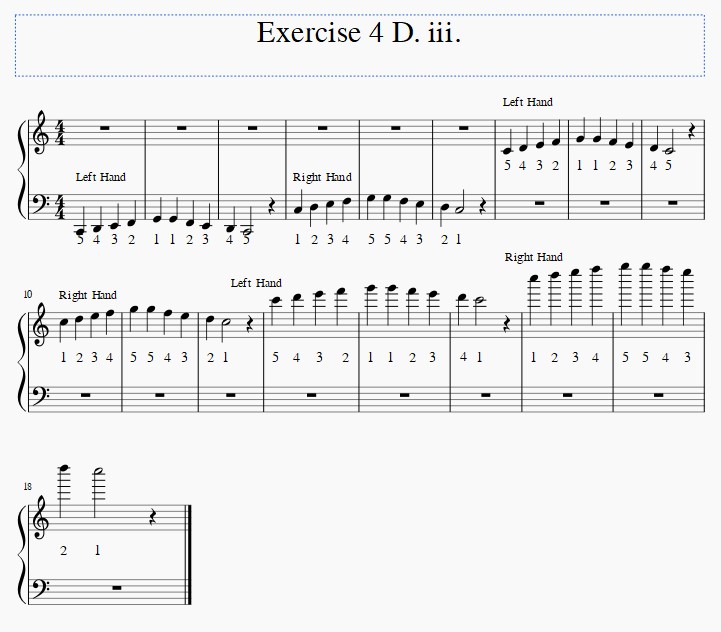


1. **FREE MELODIC IMPROVISATION in octave space using heptatonic scales (eg. Dorian) for exploration of free and spontaneous hand/finger movements**
   - 1. Participant is given visual parameters of a few octaves, only white keys with starting and ending points being identified with stickers. Ionian, Dorian, Mixolydian, and Aeolean
     2. Therapist sets timer for approximately 2 minutes
     3. Therapist begins with Bourdon and cues with drum, rhythm, or bourdon
2. **HOME –BASED EXERCISES**

- 1. Show participant how to do all exercises under # 2 and # at home with music and metronome
  2. Provide participant with castanets for home training
