# Supplementary material for: Randomized controlled trial of neurologic music therapy in Parkinson’s disease: research rehabilitation protocols for mechanistic and clinical investigations
Source: Trials. 2021 Aug 28;22:577. doi: 10.1186/s13063-021-05560-7 (PMC8403394; doi:10.1186/s13063-021-05560-7)
Supplement: Supplementary file 2 — Additional file 2. TIMP-NR research protocol. [file 13063_2021_5560_MOESM2_ESM.docx]

**TIMP-NR RESEARCH PROTOCOL**

**1. Greet participant, caregiver**

# 2. WARM UP EXERCISES


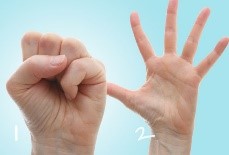
 **a. WHOLE HAND MOVEMENTS:**

1. Open and close hands; both hands moving together – 10 repetitions. Look for large opening of the hands.
2. No Cueing, participant counts repetitions on their own.


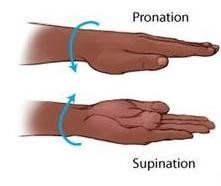


1. Pronation/supination; both hands moving together – 10 repetitions. Look for complete turns of the hands.
2. No Cueing, participant counts repetitions on their own.

#
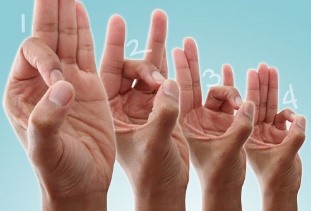
b. FINGER-THUMB OPPOSITION MOVEMENTS (TOUCHING THUMB SEQUENTIALLY WITH OTHER 4 FINGERS)

1. Verbally cue movements; both hands move together **3x without pause**
2. Remind participant to do FULL EXTENSION OF FINGERS

# c. HAND TO OBJECT MANIPULATIONS: REACHING, GRASPING, HOLDING, LIFTING OBJECTS

1. Have participant sit in front of small table with 3 different objects; a cup, a spoon, and a coin. Items will be picked up, transferred, and set down (Reach, Grasp, Lift, Transfer, Let Go and bring arm back). Verbally cue directions prior to start (no verbal cueing during task).
2. Sessions 1-5: **Midline transfer to matching side**.


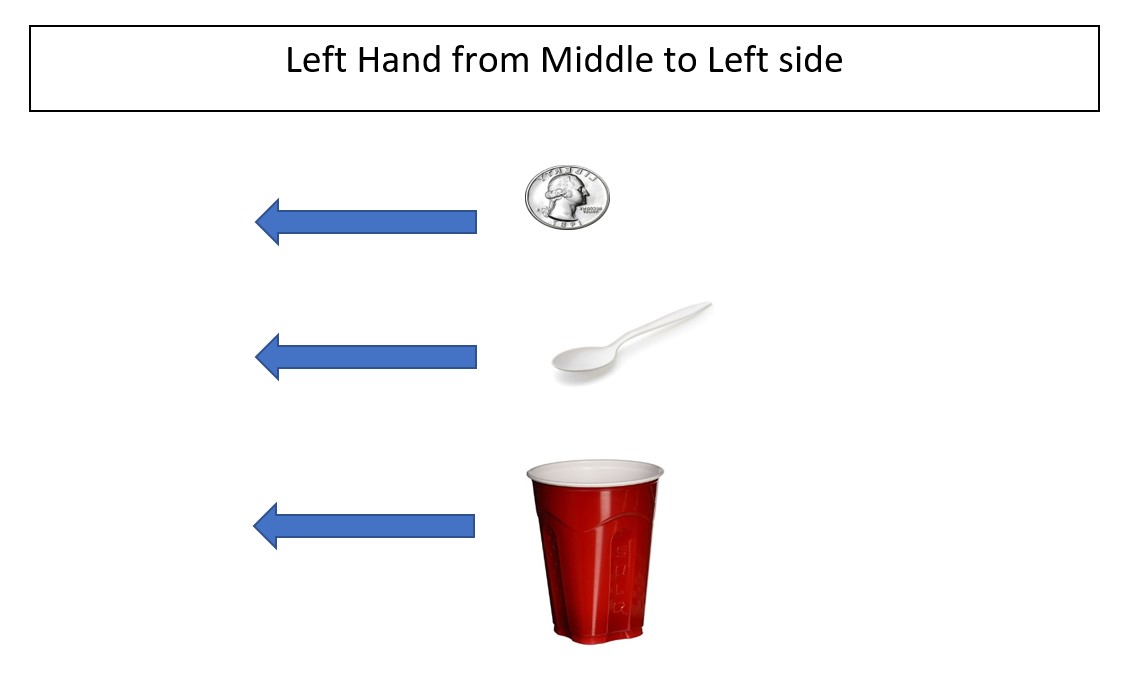

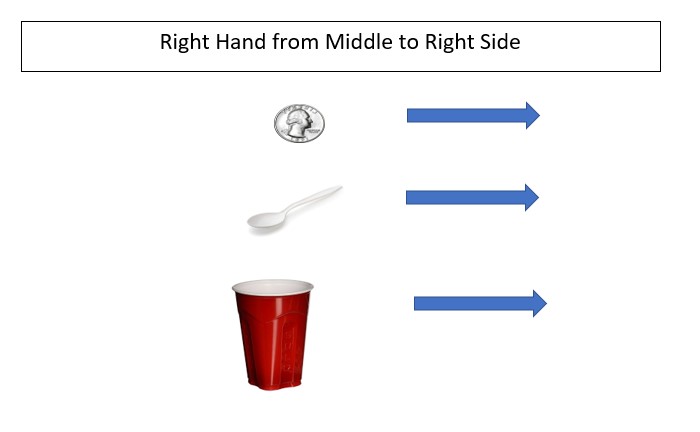


# iii. Sessions 6-10: Midline transfer to opposing side


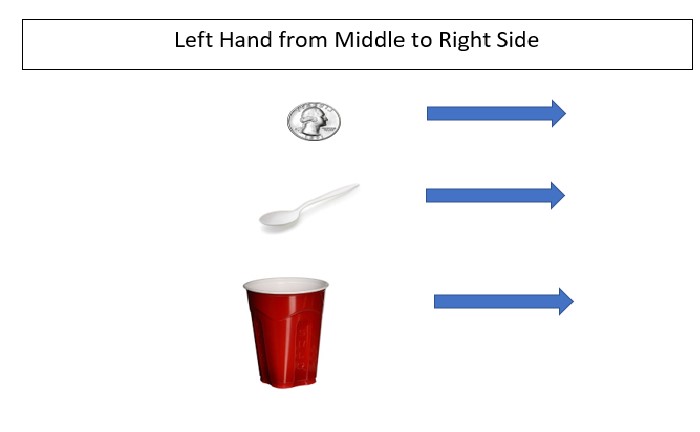

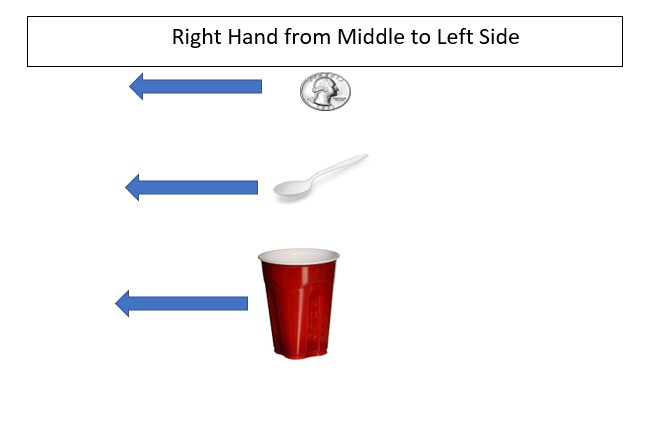


# iv. Sessions 11-15: Opposing side transfer across to matching hand side


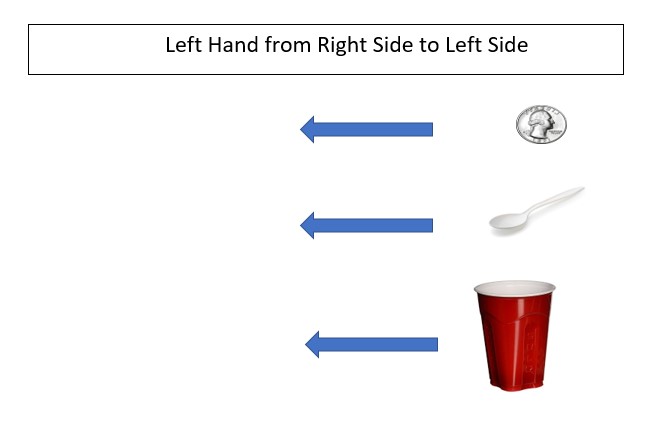

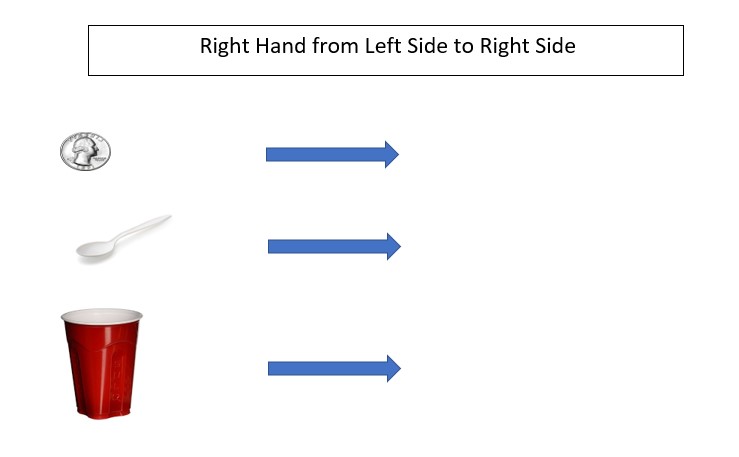


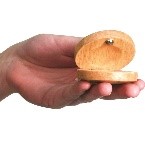


# 3. CASTANET PLAYING EXERCISES WITH MUSIC THERAPIST SINGING ALONG

1. Give participant castanets, one in each hand.
2. Have participant show you how fast they are going to play the castanets. Match the participant’s tempo.
3. Therapist sings preferred song or set of songs that total 3-4 minutes (offer opportunity for participant to also sing along).
4. Participant starts with both hands playing castanets simultaneously.
5. Halfway through therapist gives directions to switch to alternating hands

# 4. DEXTERITY EXERCISES ON KEYBOARD

**! Do not give external cues (no foot tapping/no snapping).**

**a. FINGER ADJACENT SCALES IN DIFFERENT FINGER COMBINATIONS:**

1. Have participant play scales. Repeat each exercise x1 100% success rate each hand (or more if extra time). Do not give external cues (no foot tapping/no snapping). Starting Pitch- Middle C (C4) on RH and C below middle C (C3) on LH

| RH 1-2-3-4-5, 5-4-3-2-1 | LH 5-4-3-2-1, 1-2-3-4-5 |
| --- | --- |

Right hand


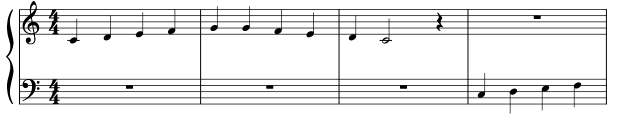


Left hand


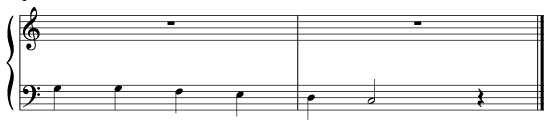


1. Have participant play arpeggios. Repeat each exercise x 1 100% success rate each hand (or more if extra time).

Starting Pitch- Middle C (C4) on RH and C below middle C (C3) on LH

| RH 1-3-5, 5-3-1 | LH 5-3-1, 1-3-5 |
| --- | --- |


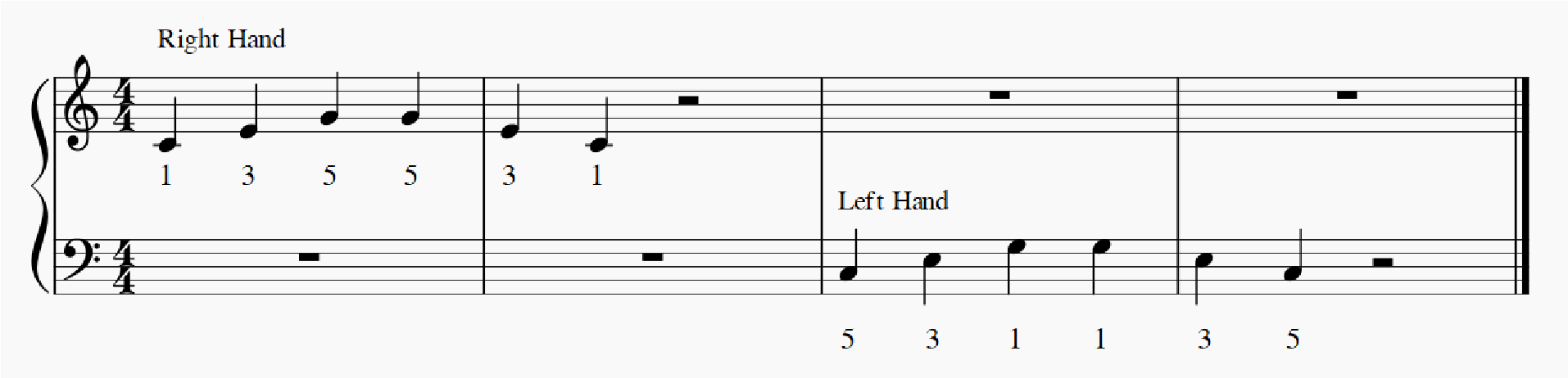


1. Have participant play alternating pattern. Repeat each exercise x 1 100% success rate each hand (or more if extra time). Starting Pitch- Middle C (C4) on RH and C below middle C (C3) on LH

| RH 1-5-2-5-3-5-4-5, 5-4-5-3-5-2-5-1 | LH 5-1-4-1-3-1-2-1, 1-2-1-3-1-4-1-5 |
| --- | --- |


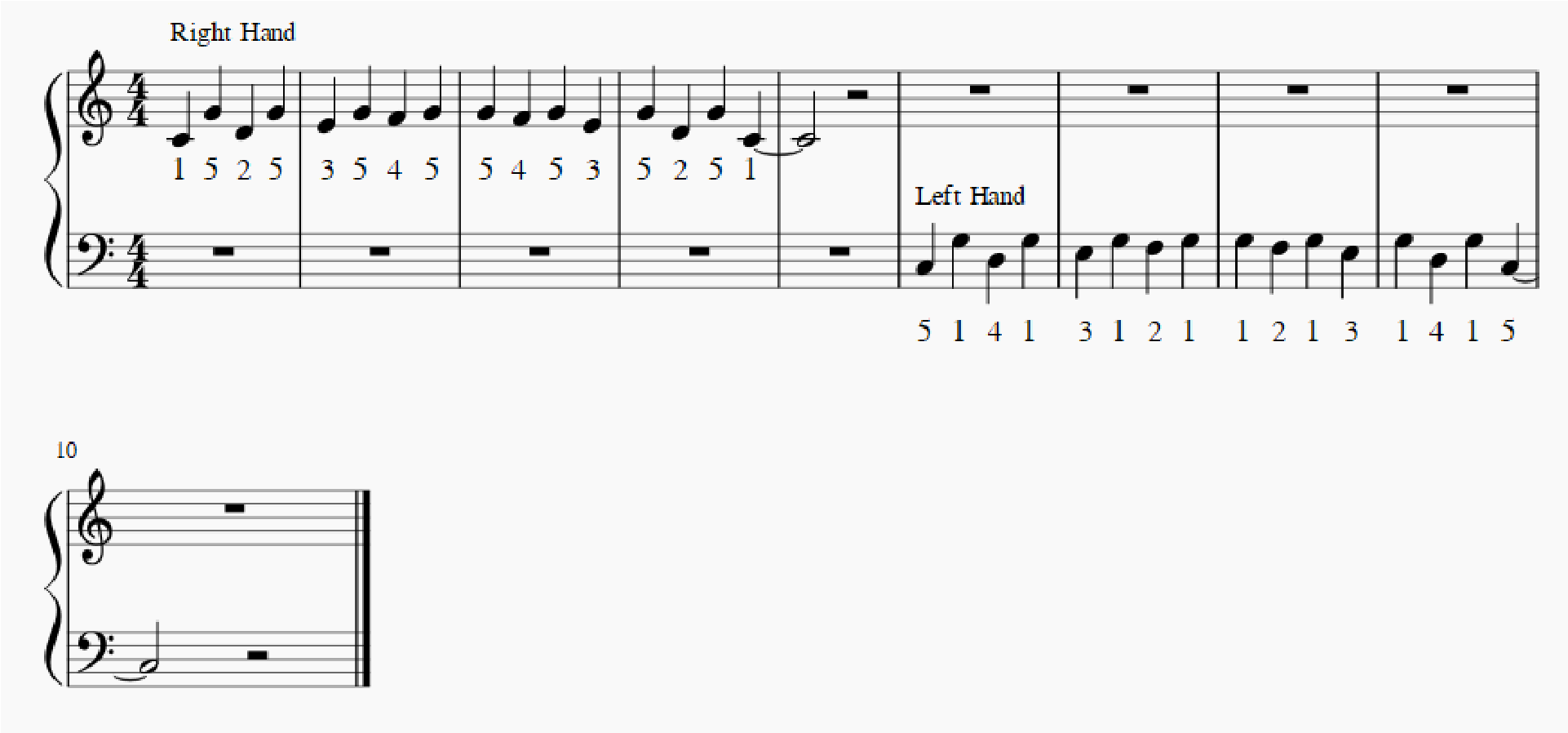


# b. SCALES PLAYING IN DIFFERENT OCTAVE REGIONS TO TRAIN FOR FLEXIBILITY AND DISTAL MUSCLE INVOLVEMENT

1. Starting Pitch RH- C6, One scale per octave, move down keyboard for 4 octaves. Starting Pitch LH- C2, One scale per octave, move up keyboard for 4 octaves.
2. Each exercise x 1 100% success rate per hand (or more if extra time).

| RH 1-2-3-4-5, 5-4-3-2-1 | LH 5-4-3-2-1, 1-2-3-4-5 |
| --- | --- |


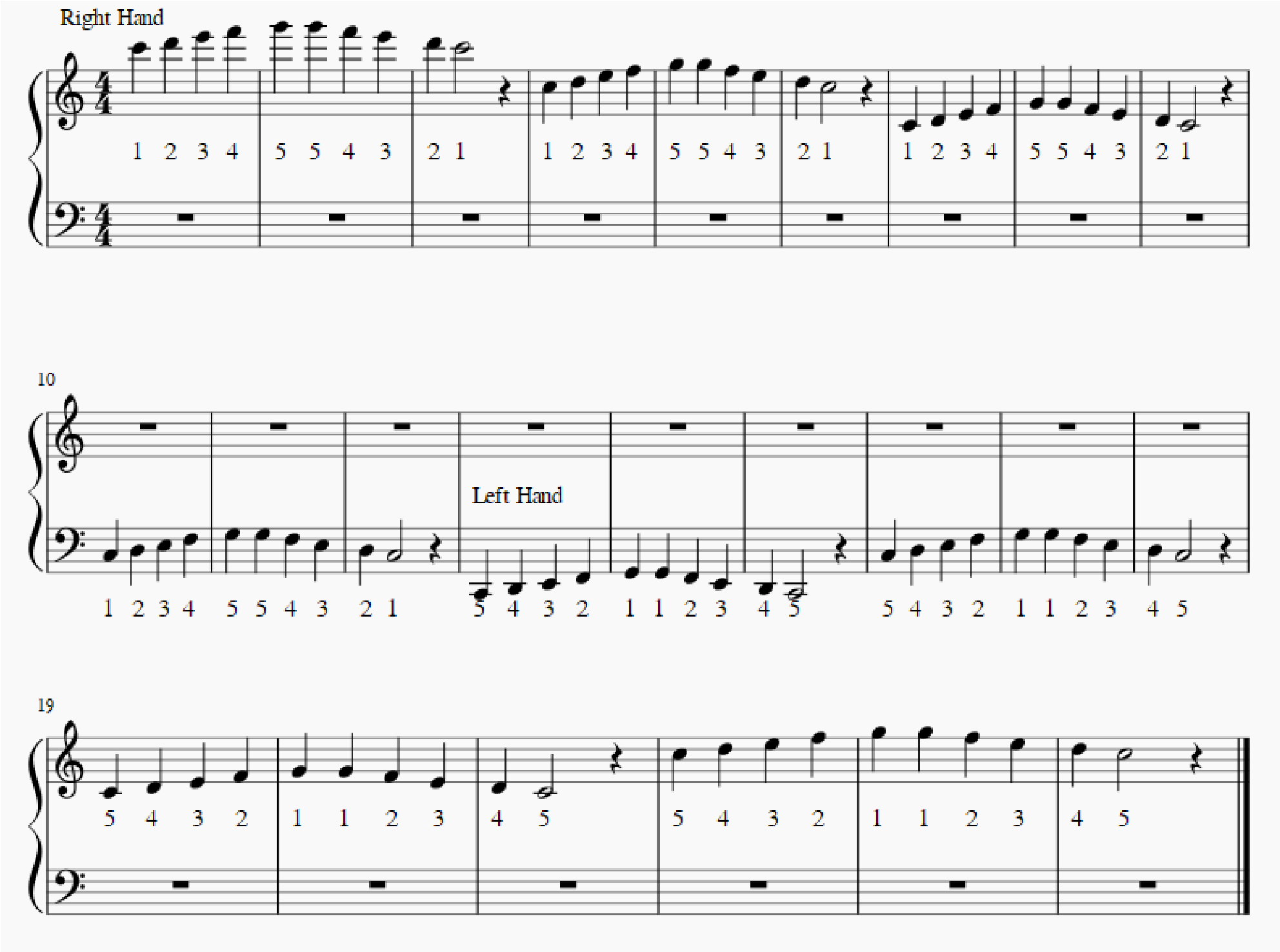


1. Each exercise x1 100% success rate per hand (or more if extra time).

Starting Pitch RH- C6, One scale per octave, move down keyboard for 4 octaves.

Starting Pitch LH- C2, One scale per octave, move up keyboard for 4 octaves.

| RH 1-3-5, 5-3-1 | LH 5-3-1, 1-3-5 |
| --- | --- |


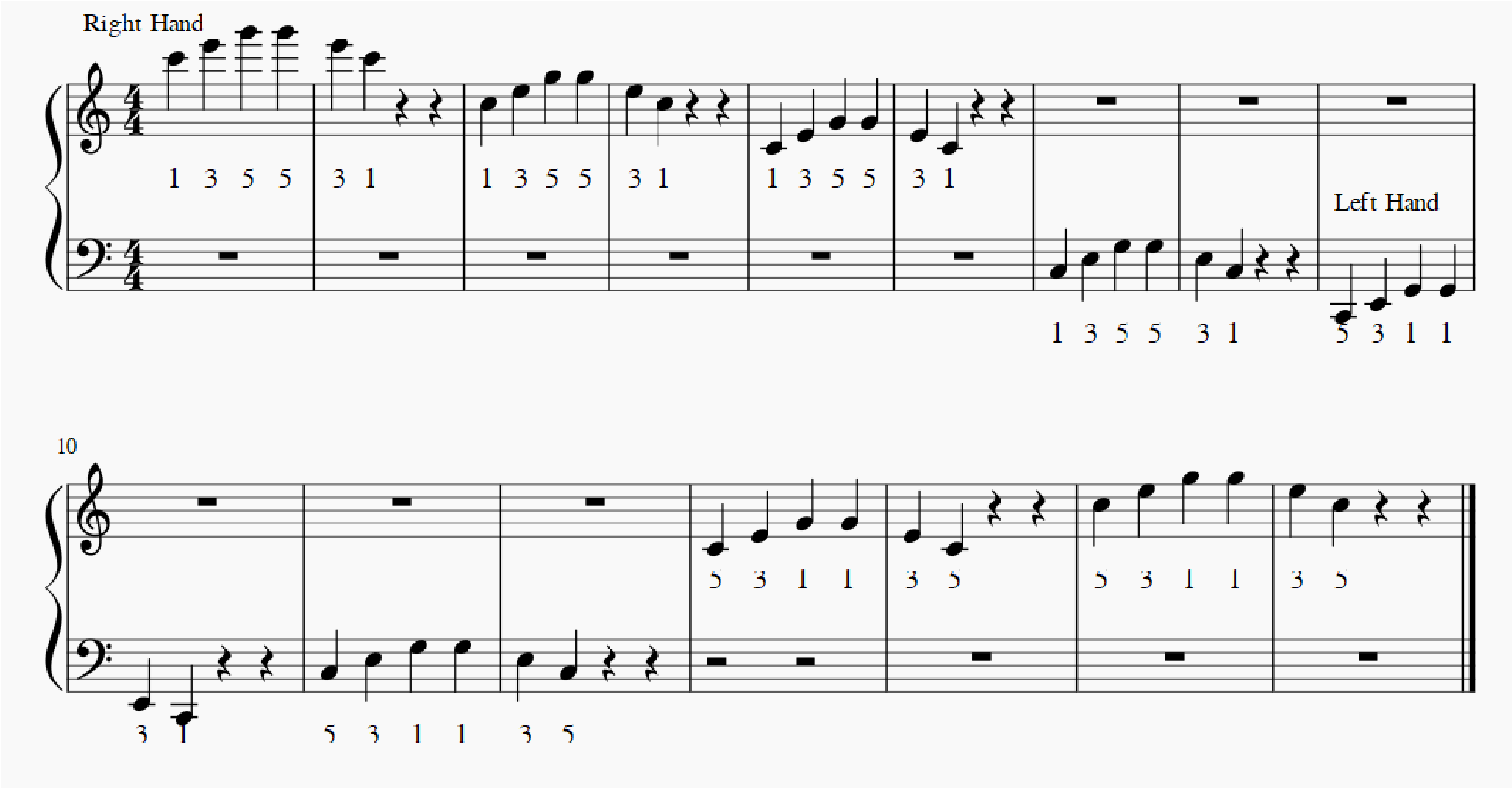


1. Each exercise x1 100% success rate per hand (or more if extra time).

Starting Pitch RH- C6, One scale per octave, move down keyboard for 4 octaves.

Starting Pitch LH- C2, One scale per octave, move up keyboard for 4 octaves.

| RH 1-5-2-5-3-5-4-5, 5-4-5-3-5-2-5-1 | LH 5-1-4-1-3-1-2-1, 1-2-1-3-1-4-1-5 |
| --- | --- |


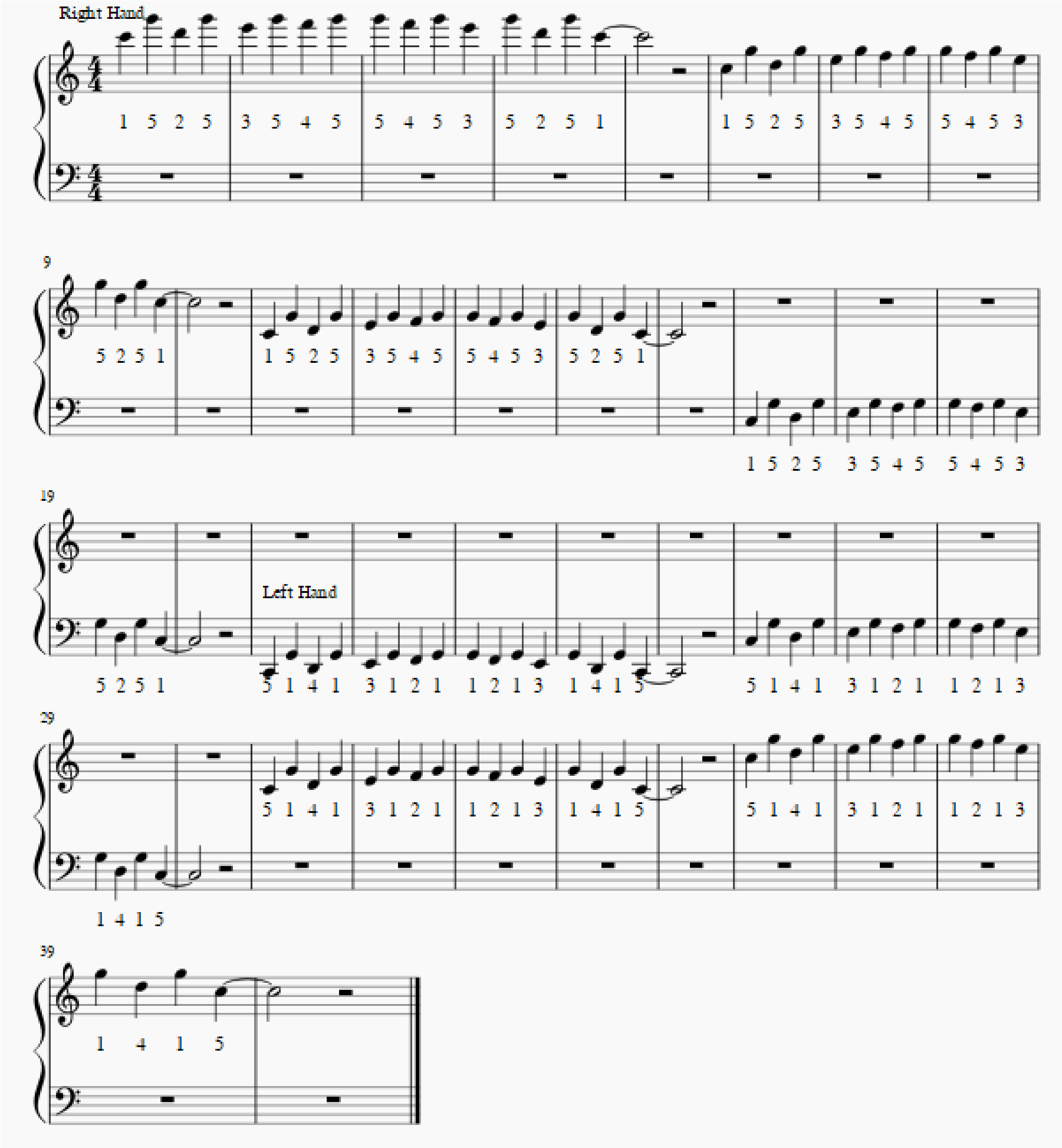


**c. SCALES PLAYING IN 3 HAND COMBINATIONS: ALTERNATING, SEQUENTIAL, AND SIMULTANEOUS:**

1. Each exercise x1 100% success rate per hand (or more if extra time).

Alternating Starting Pitch- RH C5; Starting Pitch- LH C3

| RH 1-2-3-4-5, 5-4-3-2-1 | LH 5-4-3-2-1, 1-2-3-4-5 |
| --- | --- |


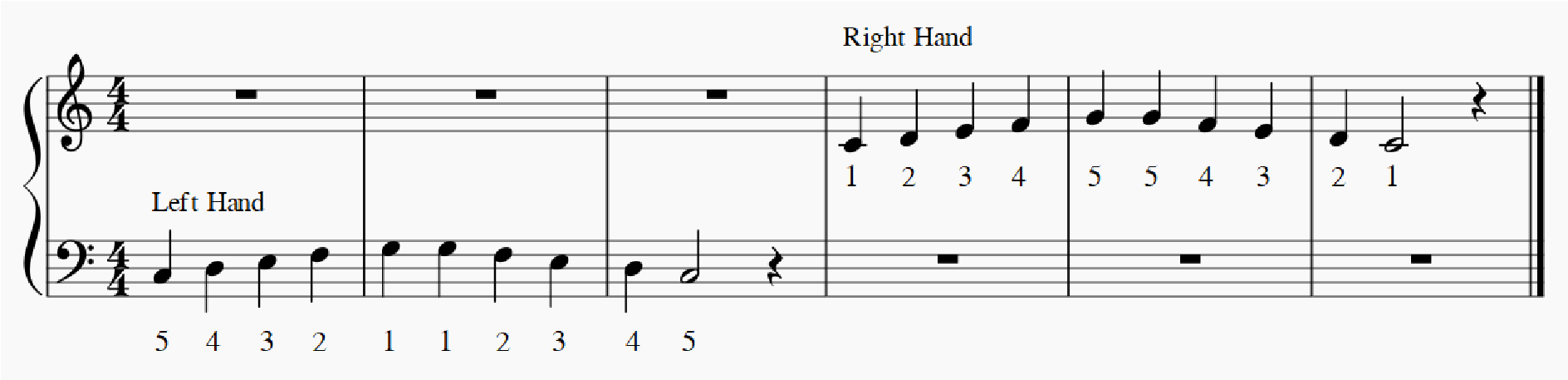


1. Each exercise x1 100% success rate per hand (or more if extra time).

Simultaneous Starting Pitch- RH C5; Starting Pitch- LH C3

| RH 1-2-3-4-5, 5-4-3-2-1 | LH 5-4-3-2-1, 1-2-3-4-5 |
| --- | --- |


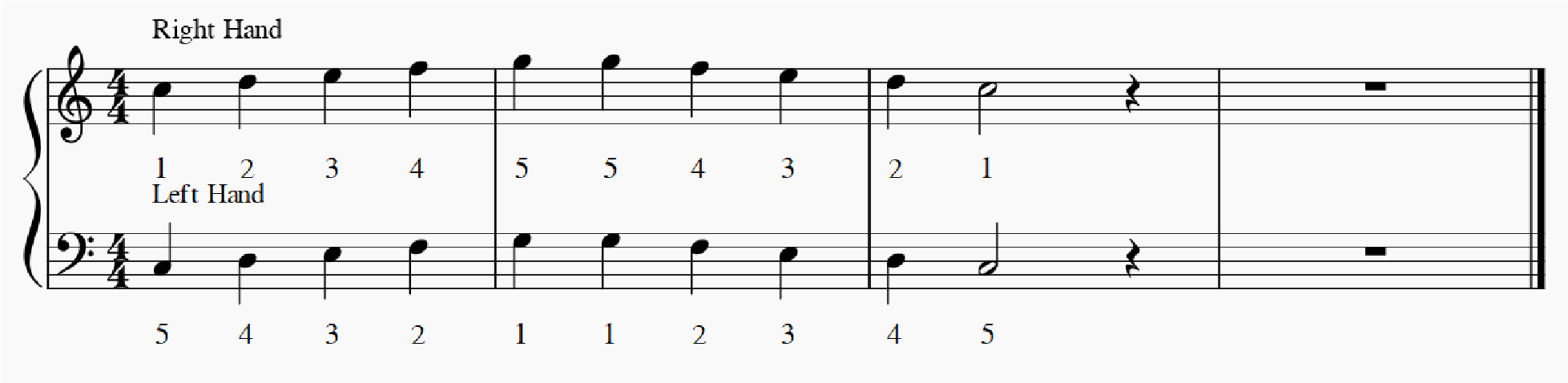


1. Each exercise x1 100% success rate per hand (or more if extra time).

Sequential Starting Pitch- LH C2, RH C3; Ending pitch- RH C7, LH C6

| RH 5-4-3-2-1, 1-2-3-4-5 | LH 1-2-3-4-5, 5-4-3-2-1 |
| --- | --- |


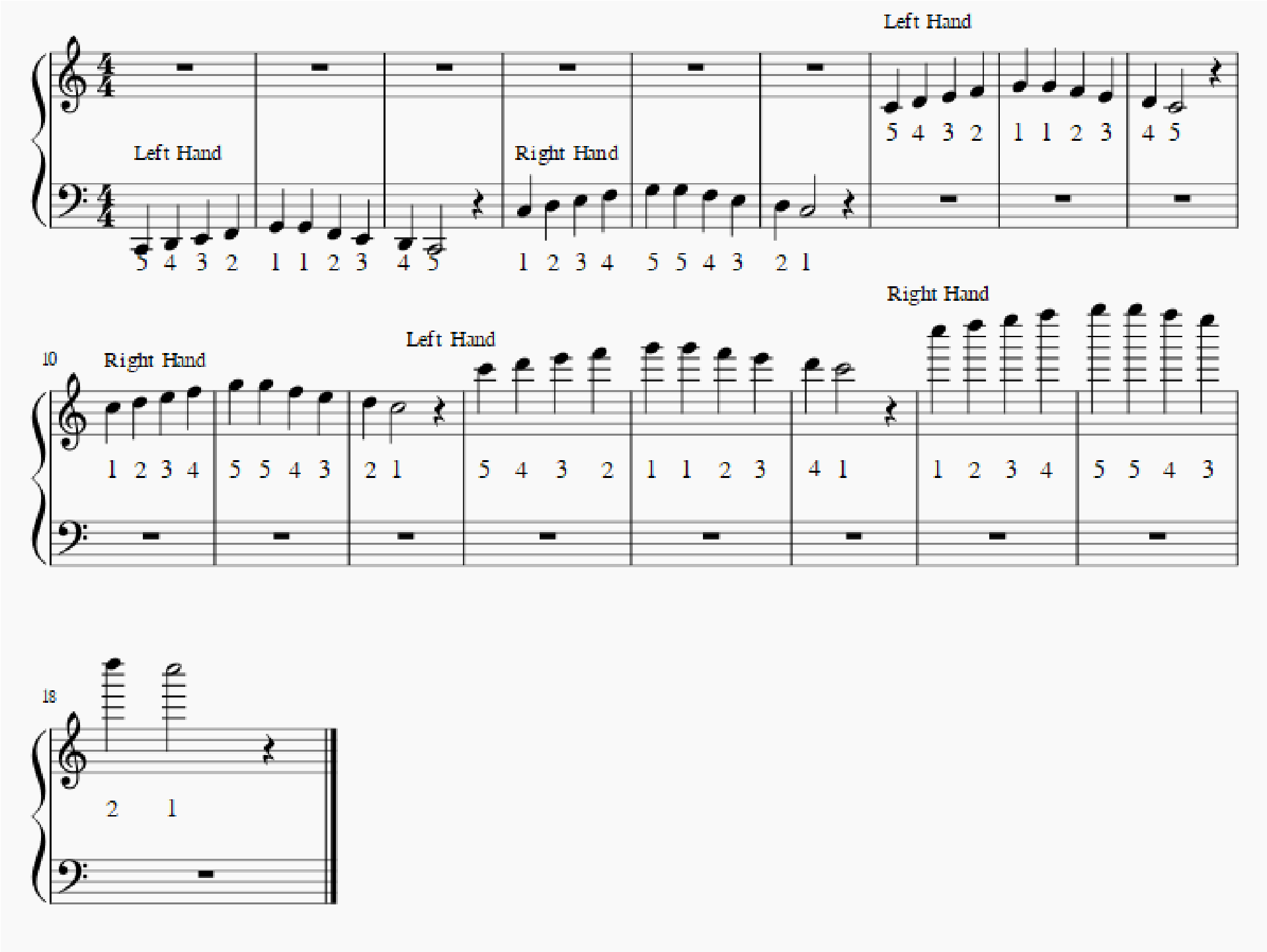


# 5. FREE MELODIC IMPROVISATION in octave space using heptatonic scales (eg. Dorian) for exploration of free and spontaneous hand/finger movements

1. Participant is given visual parameters of a few octaves, only white keys with starting and ending points being identified with stickers. Ionian, Dorian, Mixolydian, and Aeolean
2. Therapist sets timer for approximately 2 minutes
3. Therapist can give participant suggestions for improvisation.

# 6. HOME –BASED EXERCISES

Show participant how to do all the exercises under #2 and 3 at home. Encourage participant to sing along during exercise 3.

Provide participant with castanets for home exercises.
