# Supplementary material for: Randomized controlled trial of neurologic music therapy in Parkinson’s disease: research rehabilitation protocols for mechanistic and clinical investigations
Source: Trials. 2021 Aug 28;22:577. doi: 10.1186/s13063-021-05560-7 (PMC8403394; doi:10.1186/s13063-021-05560-7)
Supplement: Supplementary file 3 — Additional file 3. OT research protocol. [file 13063_2021_5560_MOESM3_ESM.docx]

**OT Research Protocol**

1. **Greet participant, caregiver**
2. **Warm Up Exercises (details on page 3) – 1 Minute Each (5 Minutes total)**
   1. Pronation/supination: both hands moving together
   2. Finger Abduction & Adduction: hands together
   3. Wrist Extension: hands together
   4. Full Fist Flexion: hands together
   5. “Duck Bill” Position: hands together

Arm Ergometer
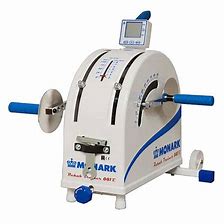


1. **Arm Ergometer (5 Minutes):**

RPM’s – Alternating: 1 minute forwards, 1 minute backwards, repeat to fulfil 5 minutes.

1. **Typing Task (5 Minutes):**

Speed and accuracy (Typingtest.com)

1. **Theraputty (5 Minutes):**

5lb; light resistive theraputty
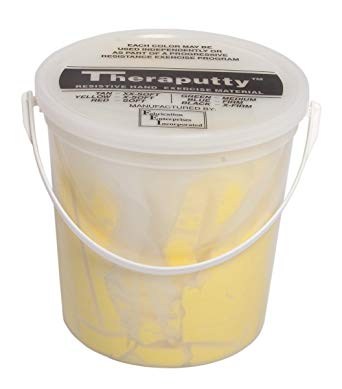


5lb container; larger mass than traditional size; bi-manual exercises focusing on rolling out into a cylindrical shape, thumb>each finger opposition, thumb lateral pinch, lumbrical position.

Every 5^th^ session, assess if the participant is able to move
onto the next level of Theraputty resistance. If they are struggling,

keep the participant on the resistance level that promotes their

best quality movement. (Tan > Yellow > Red)

Please indicate which session # which the Theraputty was changed to the next resistance level:

| Tan | Yellow | Red |
| --- | --- | --- |
| 1 |  |  |

1. **Minnesota Manipulation Activity (5 Minutes):**

Minnesota Manipulation Activity
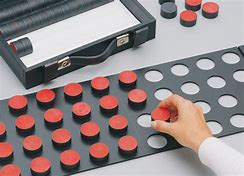


Pick up each target object one at a time, rotate palm up, and then have the other hand take the target and place it into the identified location.

1. **Handwriting (5 Minutes):**

Pre-determined paragraph; participant prints out entire paragraph in lower case letters.

1. **Slap – Tap (5 Minutes):**
   1. Alternating movement patterns.
   2. Code to follow right- and left-hand tapping

“d” = Right hand taps the table; “p” = left hand tapes the table

d p d d p p d p d d d p p p d d p

p p d d p p p d d p d p d p p d d

1. **Coloring Task (3 minutes):**
   1. For the first visit of each week, provide participant a selection of designs to choose from and colored pencils.
   2. Note which week each design was started, these will be collected at the end of each subjects’ participation.
   3. You can provide additional coloring templates if they complete the designs before the end of the week.

1. **Home-Based Exercises**

Show participant how to do all exercises under #2 at home. **Provide with paper or electronic copy of page 3.**

**Repetition Table**

| **Task** | **Week 1** | | | **Week 2** | | | **Week 3** | | | **Week 4** | | | **Week 5** | | |
| --- | --- | --- | --- | --- | --- | --- | --- | --- | --- | --- | --- | --- | --- | --- | --- |
| **Warm Up** |  |  |  |  |  |  |  |  |  |  |  |  |  |  |  |
| **Typing Task** |  |  |  |  |  |  |  |  |  |  |  |  |  |  |  |
| **MMA** |  |  |  |  |  |  |  |  |  |  |  |  |  |  |  |
| **Handwriting** |  |  |  |  |  |  |  |  |  |  |  |  |  |  |  |
| **Slap – Tap** |  |  |  |  |  |  |  |  |  |  |  |  |  |  |  |

**Warm Up/Home Exercises**


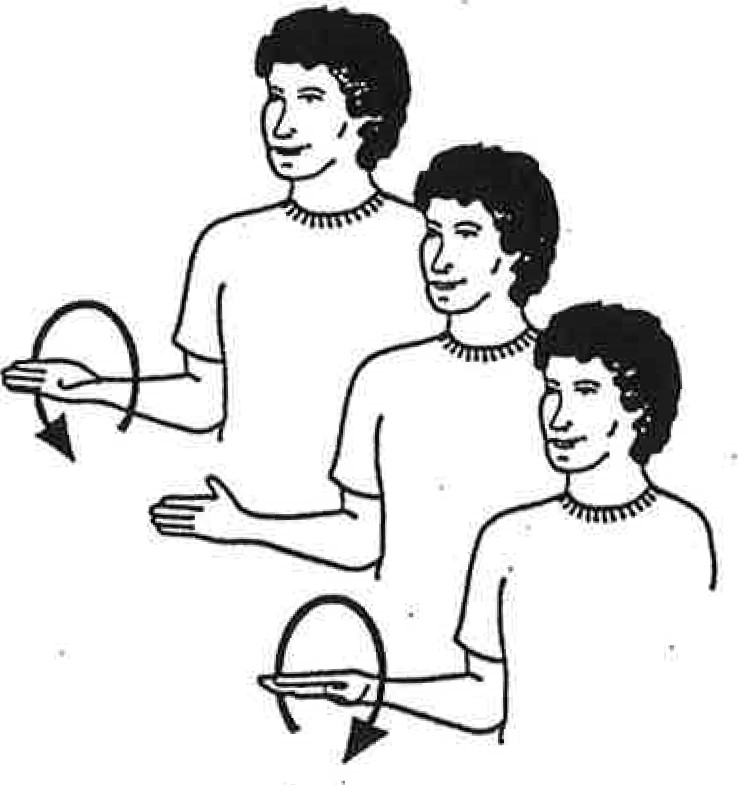


**
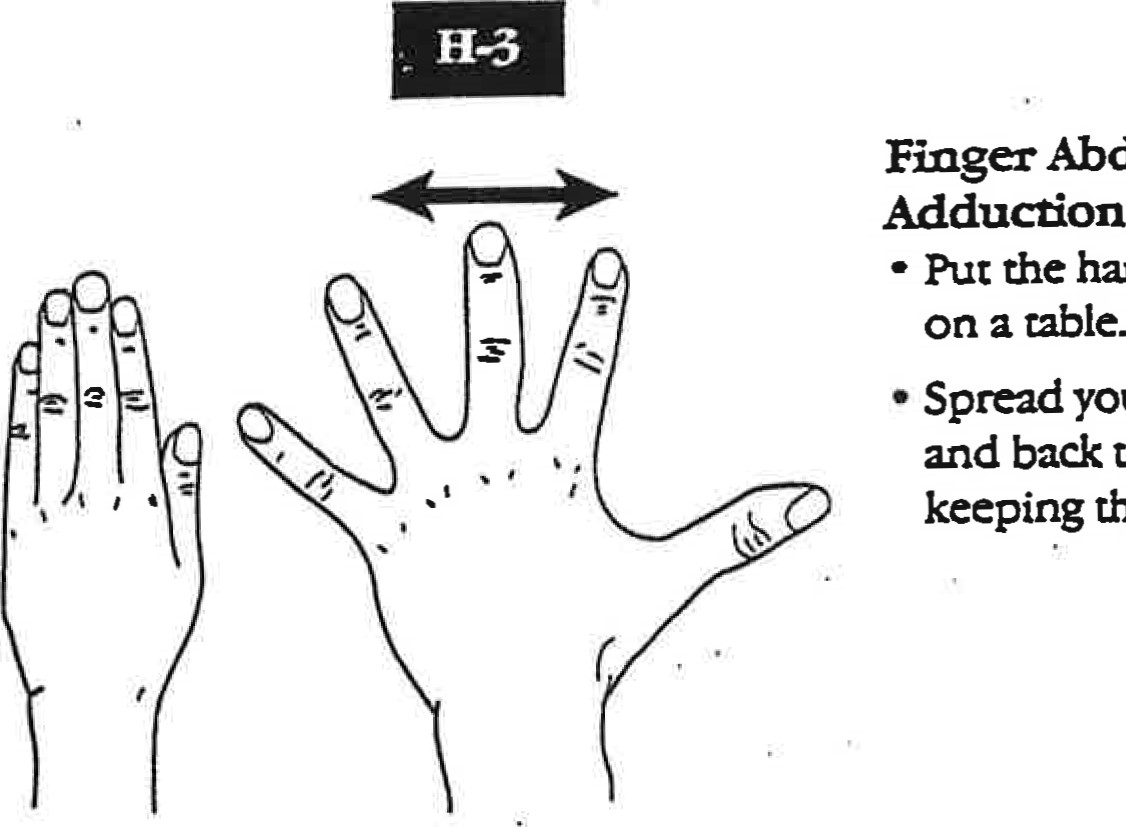
**

**Finger Abduction & Adduction**

1. Put the hand palm down on a table
2. Spread your fingers apart and then back together while keeping them on the table

**Supination/Pronation**

1. Hold your elbow bent and close to your side
2. Turn the palm towards the ceiling
3. Turn the palm then towards the floor
4. Remember to keep your elbow at your side


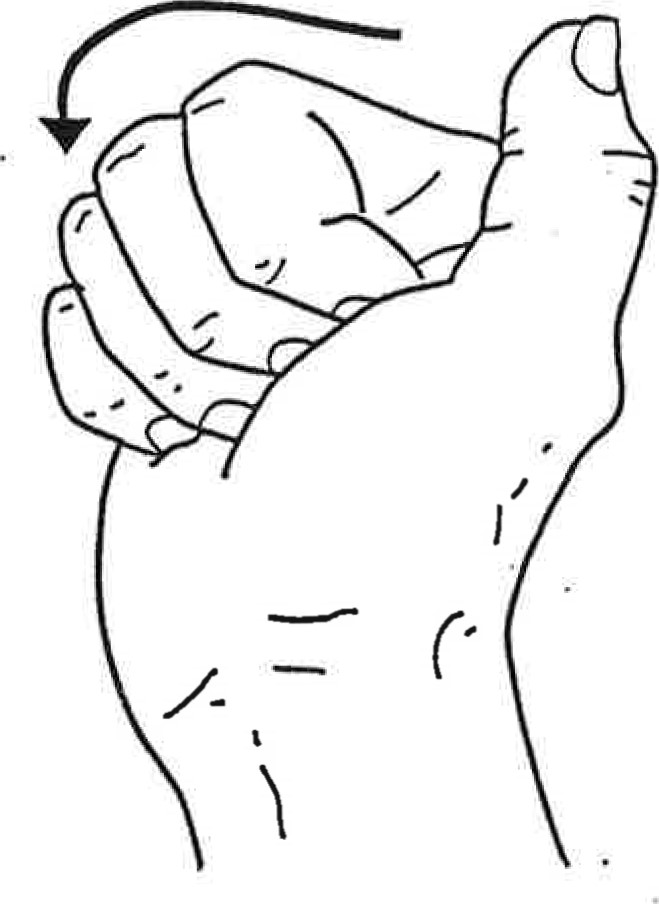

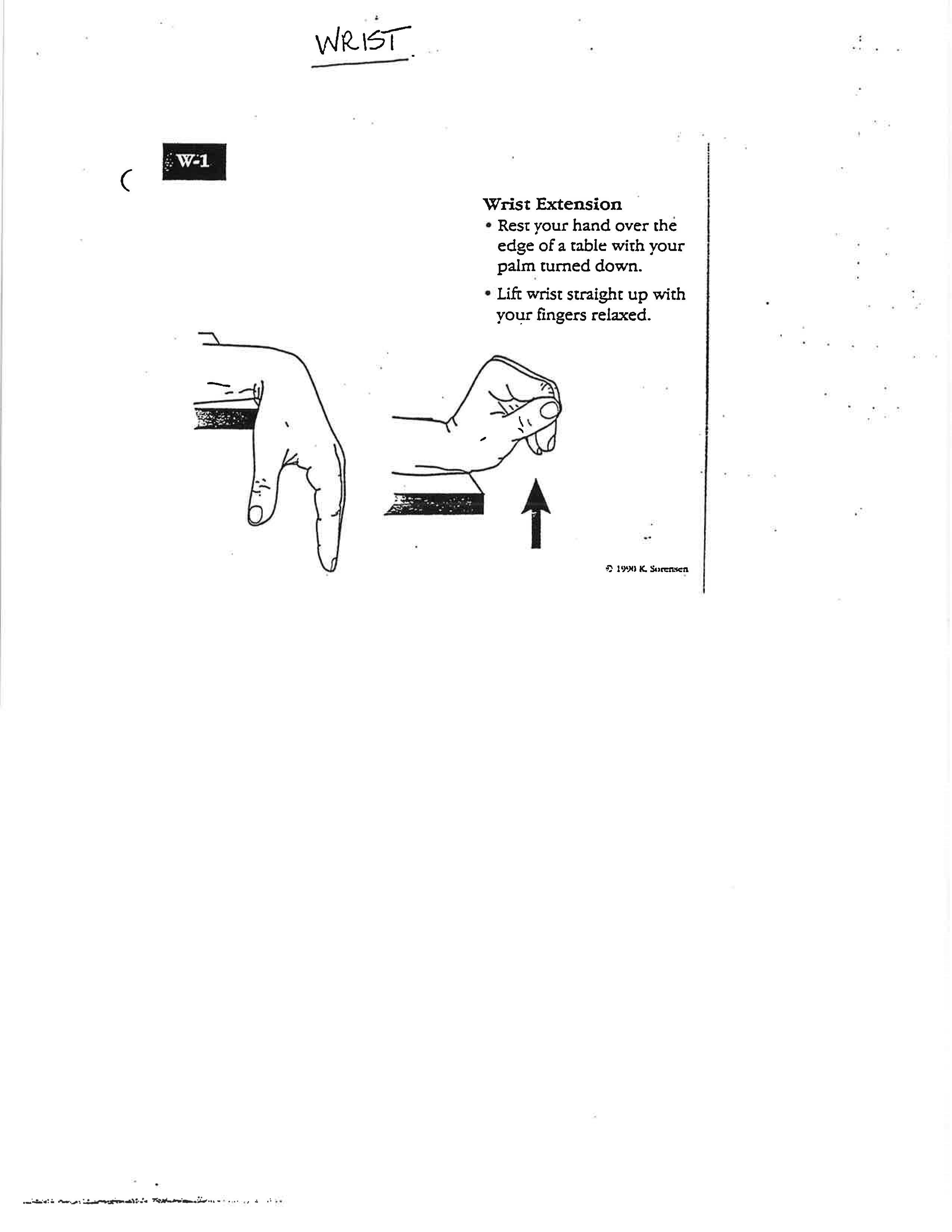


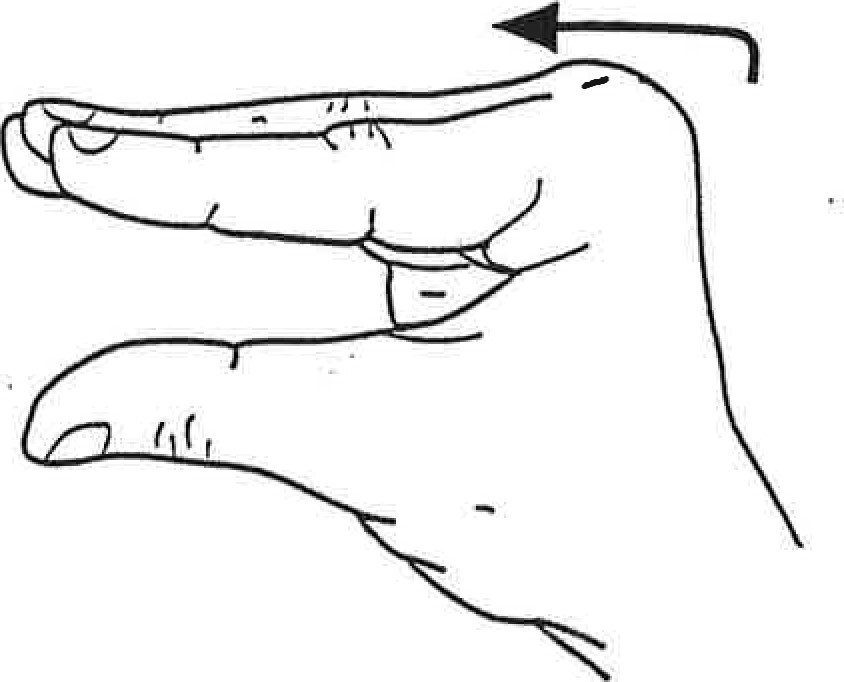


**Wrist Extension**

1. Rest your hand over the edge of a table with your palm turned down.
2. Lift wrist straight up with your fingers relaxed

**“Duck Bill” Position**

1. Bend at large knuckles while keeping fingers straight like a shelf.

**Full Fist Flexion**

1. Hold your fingers straight
2. Bend your fingers into a full fist
3. Keep thumb away from your hand
